# A Multi-Platform Draft *de novo* Genome Assembly and Comparative Analysis for the Scarlet Macaw (*Ara macao*)

**DOI:** 10.1371/journal.pone.0062415

**Published:** 2013-05-08

**Authors:** Christopher M. Seabury, Scot E. Dowd, Paul M. Seabury, Terje Raudsepp, Donald J. Brightsmith, Poul Liboriussen, Yvette Halley, Colleen A. Fisher, Elaine Owens, Ganesh Viswanathan, Ian R. Tizard

**Affiliations:** 1 Department of Veterinary Pathobiology, College of Veterinary Medicine, Texas A&M University, College Station, Texas, United States of America; 2 Molecular Research LP, Shallowater, Texas, United States of America; 3 ElanTech, Inc., Greenbelt, Maryland, United States of America; 4 Department of Veterinary Integrative Biosciences, College of Veterinary Medicine, Texas A&M University, College Station, Texas, United States of America; 5 CLC bio, Finlandsgade, Katrinebjerg, Denmark; BiK-F Biodiversity and Climate Research Center, Germany

## Abstract

This Whole Genome Shotgun project has been deposited at DDBJ/EMBL/GenBank under the accession AMXX00000000 (SMACv1.0, unscaffolded genome assembly). The version described in this paper is the first version (AMXX01000000). The scaffolded assembly (SMACv1.1) has been deposited at DDBJ/EMBL/GenBank under the accession AOUJ00000000, and is also the first version (AOUJ01000000). Strong biological interest in traits such as the acquisition and utilization of speech, cognitive abilities, and longevity catalyzed the utilization of two next-generation sequencing platforms to provide the first-draft *de novo* genome assembly for the large, new world parrot *Ara macao* (Scarlet Macaw). Despite the challenges associated with genome assembly for an outbred avian species, including 951,507 high-quality putative single nucleotide polymorphisms, the final genome assembly (>1.035 Gb) includes more than 997 Mb of unambiguous sequence data (excluding N’s). Cytogenetic analyses including ZooFISH revealed complex rearrangements associated with two scarlet macaw macrochromosomes (AMA6, AMA7), which supports the hypothesis that translocations, fusions, and intragenomic rearrangements are key factors associated with karyotype evolution among parrots. *In silico* annotation of the scarlet macaw genome provided robust evidence for 14,405 nuclear gene annotation models, their predicted transcripts and proteins, and a complete mitochondrial genome. Comparative analyses involving the scarlet macaw, chicken, and zebra finch genomes revealed high levels of nucleotide-based conservation as well as evidence for overall genome stability among the three highly divergent species. Application of a new whole-genome analysis of divergence involving all three species yielded prioritized candidate genes and noncoding regions for parrot traits of interest (i.e., speech, intelligence, longevity) which were independently supported by the results of previous human GWAS studies. We also observed evidence for genes and noncoding loci that displayed extreme conservation across the three avian lineages, thereby reflecting their likely biological and developmental importance among birds.

## Introduction

Despite the biological importance of numerous non-model and non-agricultural species worldwide, current research programs for many of these species include minimal genome-wide sequence and polymorphism data, thereby limiting the implementation of genomic approaches for addressing biological questions in these species [1]. The avian order Psittaciformes is but one example of an underserved biological group, with some genomic resources that have recently become available via completion of the Puerto Rican parrot genome (*Amazona vittata*) [2], and the Budgerigar (*Melopsittacus undulatus budgerigar*) sequencing initiative (http://aviangenomes.org/budgerigar-raw-reads/). Notably, Psittaciformes is comprised of three families: the Psittacidae (true parrots), Cacatuidae (cockatoos), and Strigopidae (New Zealand parrots) [3]. Within the Psittacidae alone, there are over 300 divergent species that display substantial geographic, phenotypic, cognitive, and behavioral variation [4]–[7], yet little is currently known about the individual genomes of these unique avian species. Relative to other avian families, Psittacidae has been estimated to contain the highest number of threatened or endangered bird species [8]–[9], thus making their study a high priority for future conservation efforts. Moreover, the conservation status of this family has strongly catalyzed research in many important biological areas including phylogenetics, population genetics, natural history, nutrition, and conservation biology [10]–[14].

To date, the most well-funded and routinely studied avian genomes provide representation from the orders Galliformes (*Gallus gallus*, chicken; *Meleagris gallopavo*, turkey) and Passeriformes (*Taeniopygia guttata*, Zebra finch) [15]–[17], with recent efforts providing some genomic insight into the Psittacidae [2] (http://aviangenomes.org/budgerigar-raw-reads/), which in conjunction with this study, are expected to be important for investigating key features of this family, such as longevity and intelligence [5]–[7], [18]. The estimated time since divergence for members of Psittaciformes and the chicken (*G. gallus*; Galliformes) is approximately 122–125 MYA, whereas Psittaciformes and Passeriformes (Zebra finch; *T. guttata*) diverged more recently (78–119 MYA; for review see http://www.timetree.org/) [19]–[20]. Importantly, the generation of new avian genome assemblies, such as those representing the flycatchers (*Ficedula* spp.) [21], Darwin’s finch (*Geospiza fortis*; http://gigadb.org/darwins-finch/), the Budgerigar http://aviangenomes.org/budgerigar-raw-reads/), and additional species of Psittacidae will provide substantial comparative and evolutionary insight into avian variation in traits such as longevity, body size, intelligence, and adaptability [4]–[7], [9], [12]–[13], [17]–[18]. Given ongoing conservation initiatives [22]–[24], natural history studies [24]–[26], and population genetics research [11], [27]–[28] for the scarlet macaw (*Ara macao*; Pscittacidae), we chose this species for genome sequencing, assembly and annotation, thus providing a new representative model for the genomic information content of large neotropical psittacines.

Herein, we hypothesized that an avian multi-platform draft *de novo* genome assembly could be rapidly generated with limited funding, and subsequently found evidence to support this hypothesis, with aspects of our final results (i.e. N50 contig size, largest contig, total sequencing cost, etc) directly compared to several well annotated and established avian assemblies [15]–[17]. In the absence of existing cDNA libraries generated from multiple scarlet macaw tissues, we also hypothesized that a custom *in silico* approach was sufficient to predict a large number of gene annotation models, theoretical transcripts, and corresponding putative proteins for the investigated species, with our final results providing strong support for this hypothesis as well. To comparatively assess the information content and organization of the scarlet macaw genome, we aligned the new draft *de novo* assembly with the chicken and zebra finch genomes, and also used comparative chromosome paints derived from flow sorted chicken macrochromosomes (autosomes 1–9 and the sex chromosomes Z and W) to establish the homologous chromosome segments in a female scarlet macaw. The results of our study directly facilitate a genomics research program for the scarlet macaw, and may also serve to enable modern genomics research in other avian species.

## Results and Discussion

### Cytogenetics and ZooFish

**Figure 1:**
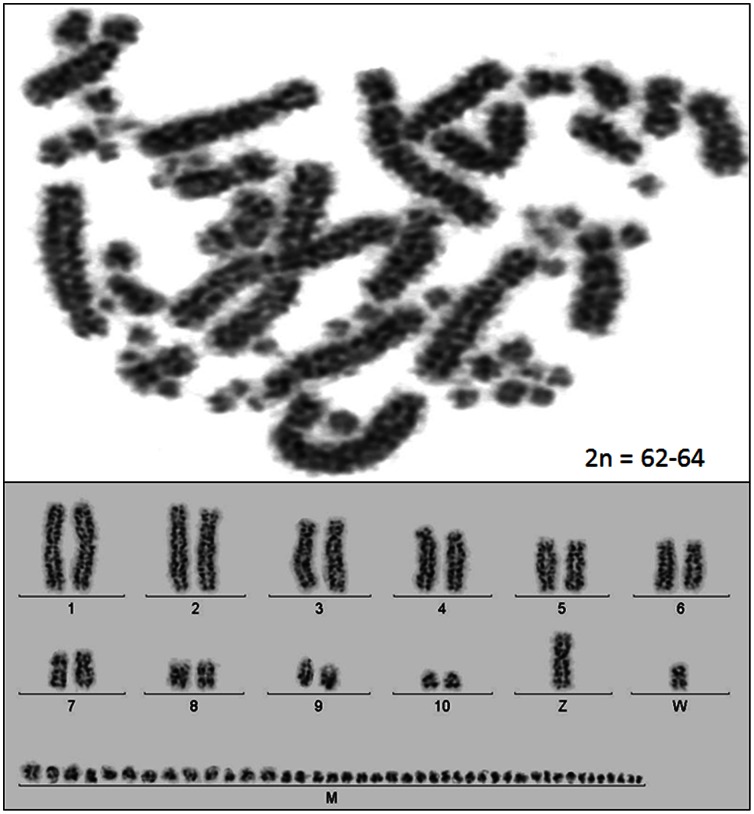
Consensus Scarlet Macaw (*Ara macao*) Karyotype. Cytogenetic analyses indicate that the scarlet macaw diploid chromosome number is 2n = 62–64, as inferred from chromosome counts of multiple cells derived from three individuals, including the sequenced female macaw (Neblina). All investigated scarlet macaws had 22 macrochromosomes, which included 10 pairs of autosomes and the sex chromosomes, and approximately 40–42 microchromosomes, the numbers of which varied due to technical reasons such as metaphase overlaps, variation in staining, and chromosome spreading.

**Figure 2:**
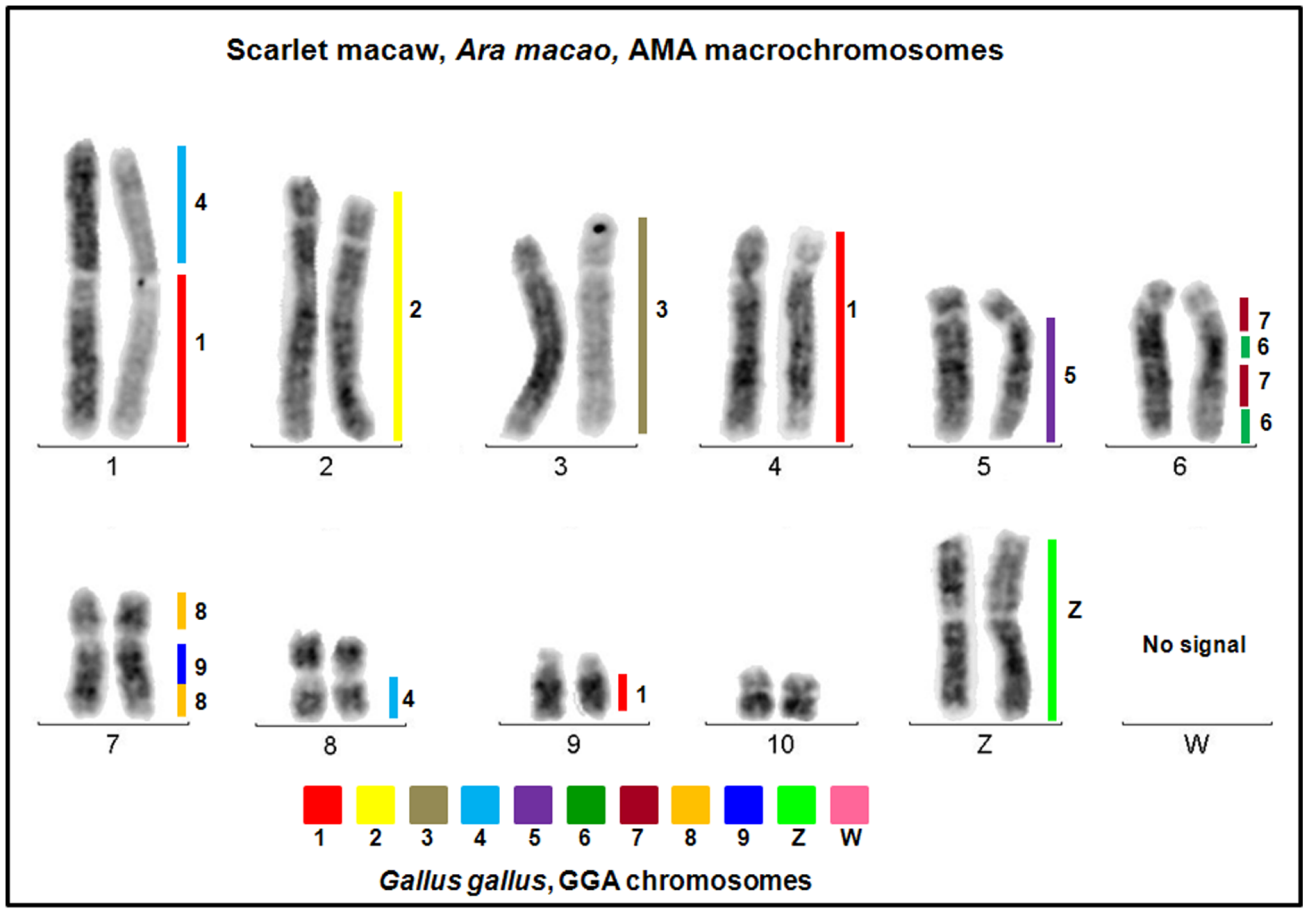
Chicken-Scarlet Macaw (*Ara macao*) Comparative Chromosome Painting (ZooFISH). Using chicken flow sorted macrochromosomes (GGA1-GGA9) as well as GGAZ and GGAW, the homologous chromosome segments of the scarlet macaw were established via fluorescent *in situ* hybridization. All flow sorted probes were validated via hybridization to chicken metaphase spreads (see Figure S1).

Cytogenetic analyses indicate that the scarlet macaw diploid chromosome number is most likely 2n = 62–64, as inferred from chromosome counts in three individuals including the female scarlet macaw selected for sequencing (“Neblina”; Figure 1). All investigated birds had 22 macrochromosomes, which included 10 pairs of autosomes and the sex chromosomes, and approximately 40–42 microchromosomes, the numbers of which varied due to technical reasons such as metaphase overlaps, variation in staining, and chromosome spreading. According to the centromere position for *A. macao* macrochromosomes (AMA): AMA1 and 8 were designated as metacentric, AMA2, 3, 4, 5, 6, 7, 10, Z and W as submetacentric, and AMA9 as acrocentric (see Figure 1, Figure 2, Figure S1). These findings are similar but not identical to the earlier description of scarlet macaw chromosomes [29] where 2n = 70 was proposed as the approximate diploid number, and the submetacentric macrochromosomes were designated as subtelocentrics. We further characterized the scarlet macaw genome by using comparative chromosome paints generated from flow sorted chicken macrochromosomes (1–9) as well as GGA-Z and GGA-W [30] to establish the homologous chromosome segments between the two avian species (Figure 2; Figure S1). All chicken chromosome paints provided discrete signals that were exclusive to their GGA chromosome of origin, as verified by their application to chicken metaphase spreads (i.e., positive controls; Figure S1). When applied to scarlet macaw metaphase spreads, individual chicken chromosome paints hybridized predominantly to a single macrochromosome pair, with the exception of GGA1 and GGA4, which hybridized to three and two scarlet macaw chromosomes, respectively (Figure 2). These results are compatible with Zoo-FISH experiments conducted between chicken and a variety of other avian species where a high degree of conserved synteny has been observed for the macrochromosomes, with the exception that GGA1 and GGA4 each tend to share homology with 2 or 3 chromosomes in other bird species [31]. As expected, no hybridization signal was detected with a GGAW probe (Figure 2; Figure S1) because, similar to the Y chromosome in mammals [32], the euchromatic sequences of the avian W chromosome are not sufficiently conserved to enable Zoo-FISH across distantly related bird species [33]–[34]. Perhaps the most interesting results obtained from our Zoo-FISH experiments are the complex rearrangements associated with scarlet macaw macrochromosomes AMA6 and AMA7 (Figure 2), which support the hypothesis that translocations, fusions, and intragenomic rearrangements are major factors associated with karyotype evolution among parrots (Psittaciformes) [35]. Moreover, the specific order of chromosome repatterning observed for AMA6, with syntenic, alternating signals derived from GGA7 and GGA6 paint probes (Figure 2), respectively, is unique in terms of the previously described rearrangements in Pscittaciformes [35], whereas the comparative configuration of AMA7 is similar to that previously described for the budgerigar (*Melopsittacus undulates*) [35]. Using a synthetic PNA probe for telomeric repeat sequences [34], we observed clear hybridization signal at the terminal ends of all scarlet macaw chromosomes (Figure S1). Moreover, no interstitial telomeric hybridization signal was detected, which is consistent with previous cytogenetic observations for the California condor, house sparrow, blue jay, and lesser adjutant stork [34], [36], but differs from observations in the chicken, Bell’s vireo, red-tailed hawk, and Inca dove, where both centric and interstitial telomeric repeat signals have been observed (for review see [34], [36]–[37]). In a final comparative analysis, we determined the location of the 5.8S, 18S, and 28S ribosomal RNA gene cluster, also known as the nucleolar organizer region (NOR), and observed three discrete scarlet macaw microchromosome pairs possess NORs (Figure S1), which was somewhat unexpected given the single microchromosome NOR previously observed for the California condor and the chicken [34]. However, relatively few avian species have been investigated for the distribution of NORs [38], and therefore, it is possible that three discrete microchromosome NORs may exist in other avian species.

### Genome Sequencing and de novo Assembly

Herein, we developed a genome sequence for “Neblina,” a female wild-caught scarlet macaw of unknown age that was originally seized by the United States Fish and Wildlife Service upon discovery of an illegal importation from Brazil. Currently, Neblina resides in Blank Park Zoo (Des Moines, IA). Unlike several other avian genomes that have benefitted from the establishment and utilization of BAC libraries, linkage and/or radiation hybrid (RH) maps, and cDNA libraries from multiple tissues [15]–[17], the draft scarlet macaw genome was derived solely from the *de novo* assembly of multi-platform next-generation sequencing data, thus representing a rapid approach to enabling efficient, large-scale genomics research programs in avian species for which classical genomic tools and resources (i.e. Maps, BACs, cDNA libraries) are limited. To maximize the recovery and precision of genome-wide sequence information in the absence of any genome maps, we assembled and analyzed the genome in two steps as follows: 1) Generation of a simple *de novo* assembly without scaffolding; 2) Generation of a *de novo* assembly with scaffolding via paired-reads. Emerging trends in avian genomics demonstrate that diverse analytical approaches have been successfully used [2], [21] (*Geospiza fortis*; http://gigadb.org/darwins-finch/; http://aviangenomes.org/budgerigar-raw-reads/) to deliver new avian genome assemblies, with this study further supporting this concept, but also pointing out some limitations in genome characterization, as a function of not generating both a simple *de novo* and scaffolded genome assembly.

Sequence data generated and utilized for this assembly were derived from three next-generation sequencing procedures as follows: Roche 454 GS-FLX with Titanium Chemistry (Roche; Branford, CT), Illumina Genome Analyzer IIx (GAIIx; v5 Chemistry; Illumina Inc.; San Diego, CA), and the Illumina HiSeq 2000 (v2 Chemistry; Illumina Inc.; San Diego, CA). Using knowledge of avian genome size (nuclear DNA content, C-value) derived from comparative flow cytometry [39] in conjunction with physical knowledge of several modern avian genome assemblies (i.e., size; base pairs) [15]–[17], we estimated the scarlet macaw nuclear genome to be approximately 1.11–1.16 Gigabase pairs (Gbp) in size. Notably, this estimate does not fully account for the apparent lack of completeness associated with existing avian genome assemblies (i.e., collapsed repeats), but does provide a useful benchmark for determining whether the overwhelming majority of the scarlet macaw genome was captured via *de novo* assembly. Collectively, more than 426 million trimmed sequence reads derived from four different sequencing libraries were used in the assembly process (Table 1), which yielded ≥26X theoretical genome coverage (1.11–1.16 Gbp) as input data for the multi-platform assembly. Based on the estimated genome size for scarlet macaw, these data represent >1X genome coverage in Roche 454 long reads, and ≤25X coverage in total Illumina reads (GAIIx and HiSeq 2000; see Table 1).

**Table 1:** Summary of Roche 454 Titanium and Illumina sequence data used for *de novo* assembly of the scarlet macaw genome.

Data Source	Total Reads^1^	Library Type	Insert Size^2^ Paired Dist. (bp)^3^	Average Read Length (bp)^4^
Roche 454	4,489,636	Random Shotgun	500–600^2^	301
Illumina GA IIx	59,090,507	Small Insert Paired End	250–450^3^	116
Illumina HiSeq	132,052,204	Small Insert Paired End	250–450^3^	93
Illumina HiSeq	116,445,199	Mate Pair (Small)	1100–2700^3^	48
Illumina HiSeq	114,034,657	Mate Pair (Medium)	4000–5700^3^	47

1 Total usable reads after quality and adapter trimming (n = 426,112,203).

2 Targeted fragment population after nebulization of high molecular weight genomic DNA.

3 Range of observed paired distances for each Illumina sequencing library.

4 Reflects the averages for quality and adapter trimmed reads.

**Figure 3:**
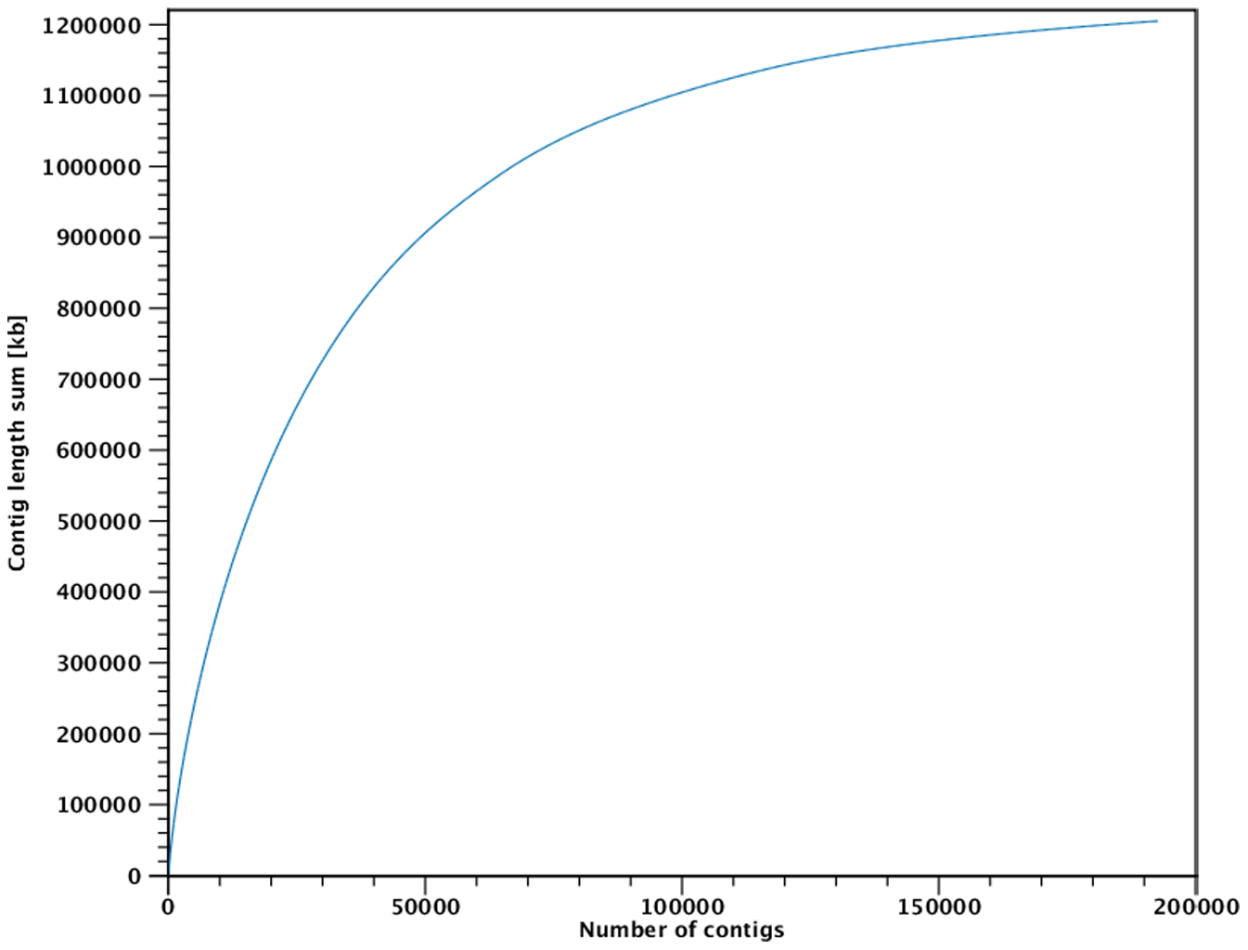
Relationship Between Total Contig Length (Kbp) and Total Contig Number for the Scaffolded Scarlet Macaw (*Ara macao*) Genome (SMACv1.1). The y-axis represents total contig length, expressed in kilobase pairs (Kbp), whereas the x-axis represents the total number of scaffolded contigs. Based on the estimated size of the scarlet macaw genome (1.11–1.16 Gbp), ≥90% of the assembled genome was captured within approximately 95,000 contigs.

We utilized 426,112,203 trimmed sequence reads in a hybrid *de novo* assembly that was conducted using the CLC Genomics Workbench (v4.8, 4.9; Finlandsgade, Dk). Details regarding the multi-step assembly procedure, including specific workflows that reduce the potential for contig misassembly, are described in the Methods. Collectively, our first-generation simple *de novo* assembly (SMACv1.0) contained 1.035 Gbp distributed across 282,983 unscaffolded contigs with N50 size of 6.37 Kbp (kilobase pairs; Table 2; Table S1; Genbank Accession AMXX00000000), which is similar to features of the unscaffolded Puerto Rican parrot genome [2]. Thereafter, and for comparison, we also generated a second *de novo* genome assembly (CLC Genomics Workbench v4.9) which included the addition of a scaffolding algorithm implemented during the final steps of the assembly process. Briefly, the scaffolding procedure used paired reads spanning two contigs to estimate the distance between the contigs, and also to determine their relative orientation. Scaffolding was performed using a greedy approach in which the smallest gaps between contigs were closed first during an iterative process, with scaffolding taking advantage of both paired-end and mate-pair reads, but with strict enforcement of the specified paired distances and read orientations (see Methods). Our first generation scaffolded *de novo* genome assembly (SMACv1.1) contained 1.205 Gbp (including gaps; N’s) distributed across 192,790 contigs, with a N50 contig size of 15.97 Kbp (Table 2; Table S2; Genbank Accession AOUJ00000000). Collectively, ≥90% of the assembled genome was captured within 95,000 contigs (Figure 3). We use the term “contigs” in relation to the final products of the scaffolded genome assembly because not every genomic sequence contig was actually joined to another via read-based scaffolding. Altogether, 140,453 (72.9%) final contigs were longer than 1 Kbp, and the largest scaffolded *de novo* contig assembled was 177,843 bp (Table 2). These results are similar to those achieved for the Puerto Rican parrot [2] and domestic turkey [16]. Based on the estimated size of the scarlet macaw genome (1.11–1.16 Gbp), it is apparent that the vast majority of the genome was captured (Table 2). Summary and comparative data involving major characteristics of the simple and scaffolded *de novo* genome assembly for the scarlet macaw are presented in Table 2, which includes a comparison with the initial releases of two established and well annotated avian genomes.

**Table 2:** Summary data for the scarlet macaw first-generation draft *de novo* genome assembly with comparison to the initial turkey and chicken genome assemblies.

Genome Characteristics	Scarlet Macaw 1.0^1^	Scarlet Macaw 1.1^2^	Turkey 2.01	Chicken 2.1
Total Contig Length (without gaps)	1.035 Gbp	0.997 Gbp	0.931 Gbp	1.047 Gbp
Total Contigs >1 Kb^3^	214,754^3^	140,453^3^	128,271	98,612
N50 Contig Size	6,366 bp	15,968 bp	12,594 bp	36,000 bp
Largest Contig	87,225 bp	177,843 bp	90,000 bp	442,000 bp
Contig Coverage^4^	16x^4^	13x^4^	17x	7x
Cost of Sequencing (M = million)	< $0.034M	< $0.034M	< $0.250M	> $10M

1 SMACv1.0 is unscaffolded. The cost of sequencing also reflects all library costs.

2 SMACv1.1 is scaffolded based on paired-reads and is 1.205 Gbp including gaps.

3 SMACv1.0 = 282,983 total contigs; SMACv1.1 = 192,790 total scaffolded contigs.

4 Median value of average coverage across all genomic contigs.

To evaluate the concordance between the two scarlet macaw assemblies, we mapped the simple *de novo* contig sequences onto the scaffolded assembly using two procedures: 1) Utilization of the large gap read mapper within the CLC Genomics Workbench (v4.9), which was limited to mapping sequences ≤7,999 bp (n = 251,604); and 2) Implementation of the blastn algorithm for sequences ≥8,000 bp (n = 31,379). Altogether, we mapped 263,777 simple *de novo* contigs (93.2%) onto the new scaffolded genome, with >62% that displayed ungapped read mappings. However, the total simple *de novo* contigs which could be mapped onto the scaffolded assembly is actually underestimated by 4.9%, since 13,875 contig mappings were considered invalid by the CLC large gap read mapper. Invalid mappings resulted from multiple nonconsecutive alignments for discrete segments of each contig, and/or the presence of inverted internal segments relative to the scaffolded reference genome. Examination of these and other discrepancies (i.e., gaps) provided evidence for collapsed repeats as well as heterozygosity within some repetitive and nonrepetitive regions (i.e., insertion/deletion). A reference table summarizing the genomic positions of all mapped simple *de novo* contigs within the scaffolded assembly was generated and is available for reference (Table S3).

### Comparative Genome Alignment, Predicted Repeat Content, and SNP Prediction

Initially, we aligned 99.97% of the scarlet macaw simple *de novo* contigs (SMACv1.0; n = 282,904/282,983 contigs) to the available chicken reference genome (*G. gallus* 2.1) via blastn, with median genome-wide summary statistics derived from the top E-value contig hits as follows: E-value = 3e-112, percent identity = 74%, and alignment length = 759 bp (See Table S4). Across all aligned scarlet macaw contigs, 38.6% produced a unique alignment to a single chicken chromosome (see Methods). Examination of the SMACv1.0 blastn alignments (E-value top hits) across all chicken chromosomes revealed relatively stable levels of nucleotide-based divergence, with alignments to GGA16 and GGAZ demonstrating the highest (Median = 80.13%; Mean = 79.45%) and lowest (Median = 73.21%; Mean = 74.77%) percent identities, respectively (Table S4). Similarly, 99.98% of the scarlet macaw simple *de novo* contigs (SMACv1.0; n = 282,915/282,983 contigs) were also aligned to the available zebra finch reference genome (*T. guttata* 1.1, 3.2.4 assembly), with median genome-wide summary statistics derived from the top E-value hits as follows: E-value = 2e-177, percent identity = 75%, and alignment length = 1030 bp. Altogether, 116,234 simple *de novo* contigs (41%) produced a unique alignment to a single zebra finch chromosome (see Methods). Investigation of all SMACv1.0 contig alignments across each zebra finch chromosome also revealed relatively uniform levels of nucleotide-based divergence (i.e., median blastn percent identities), with the exception of alignments to LG5 and TGU16, which demonstrated the highest (Median = 83.87%; Mean = 81.72%) and lowest (Median = 71.21%; Mean = 73.04%) percent identities, respectively (Table S4).

In general, similar results were also observed for comparative alignments involving the scaffolded scarlet macaw genome assembly (SMACv1.1), with median genome-wide summary statistics derived from the top E-value blastn hits against the chicken (newest release, *G. gallus* 4.0) and zebra finch genomes (*T. guttata* 1.1, 3.2.4) as follows: *G. gallus* E-value = 2e-110, percent identity = 74%, and alignment length = 707 bp; *T. guttata* E-value = 1e-163, percent identity = 75%, and alignment length = 759 bp. For both comparative alignments >99.94% of the total scaffolded contigs (n = 192,790) were aligned to the chicken and zebra finch reference genomes, with the majority of all SMACv1.1 contigs producing 5 total hits or less in both analyses (Table S5). For comparative alignments between SMACv1.1 and the chicken reference genome, the lowest percent identities were observed for GGA1 (Median = 73.63%; Mean = 74.84%) and GGAZ (Median = 73.72%; Mean = 75.44%), whereas the highest were observed for GGA16 (Median = 80.46%; Mean = 81.00%). A similar trend was also observed for alignments between SMACv1.1 and the zebra finch reference genome, with the lowest percent identities corresponding to TGUZ (Median = 73.85%; Mean = 75.04%), and the highest observed for TGU16 (Median = 80.60%; Mean = 80.39%). Detailed comparative alignment data between SMACv1.1 and the well-established genomes of the chicken and the zebra finch are provided in Table S5.

The minimum estimated repetitive DNA content for the scarlet macaw genome was approximately 5.2%, as predicted by RepeatMasker, but differed substantially between the two assemblies (Table 3). Specifically, the addition of “N’s” spanning gaps within the scaffolded genome essentially precluded the detection of many repetitive sequences (Table 3), with the implication being that the scaffolded assembly underestimates the true (i.e. minimum) genome-wide repetitive content. Estimates for the minimum genome-wide repetitive content were derived from a two-stage composite analysis employing both the chicken and zebra finch repeat libraries, with results obtained for the simple *de novo* assembly (SMACv1.0) being similar to that reported for the turkey genome (6.94%) [16], but lower than corresponding estimates for both the zebra finch and chicken [15], [17]. The vast majority of the predicted repeat content for the scarlet macaw consists of interspersed repeats, of which most belong to four groups of transposable elements including SINEs, L2/CR1/Rex non-LTR retrotransposons, retroviral LTR retrotransposons, and at least three DNA transposons (hobo Activator, Tc1-IS630-Pogo, PiggyBac). A summary of the major repetitive content predicted throughout the scarlet macaw genome is presented in Table 3. It should also be noted that after first masking the scarlet macaw genome (SMACv1.0, SMACv1.1) using only the chicken repeat library, very few additional repeats were subsequently identified (≤0.19%) during stage two of our analysis when the zebra finch repeat library was used to scan the masked scarlet macaw genome. The underlying reason for this was that utilization of either the chicken or zebra finch repeat libraries during the first stage of a composite analysis largely results in the discovery of the same scarlet macaw repetitive sequences. While repeat sequences are generally considered to be some of the most rapidly evolving sequences within the genome, it is clear from our results that many repeat elements and their respective sequences are conserved between the two highly divergent reference genomes (i.e., chicken, zebra finch) [15], [17] and the scarlet macaw, which suggests a tangible level of genome stability despite ≥78 MY since divergence (see http://www.timetree.org/) [19]–[20]. Notably, a similar level of stability was previously suggested between the turkey and chicken genomes following analyses of repetitive content [16]. Interestingly, one common feature of the scarlet macaw, chicken, turkey, and zebra finch genomes is the high proportion of LINE-CR1 interspersed repeats with respect to the total estimated repetitive content [15]–[17]. Additionally, a comparative analysis of known repetitive elements and their predicted genome-wide copy numbers revealed that the scarlet macaw possesses more SINE elements than the chicken or zebra finch [15], [17]. Detailed descriptions of all repeats predicted using RepeatMasker are available in Table S6 (SMACv1.0) and Table S7 (SMACv1.1).

**Table 3:** Major repetitive content predicted by RepeatMasker within the scarlet macaw first generation *de novo* genome assembly (SMACv1.0, SMACv1.1).

Repeat Type Predicted	Total Elements^1^	Total bp (% of Genome)^1^	Total Elements^2^	Total bp (% of Genome)^2^
SINEs	6,741	834,386 (0.08%)	6,612	816,297 (0.07%)
LINEs (L2/CR1/Rex)	189,424	35,772,793 (3.46%)	156,131	32,227,015 (2.67%)
LTR Retroviral	41,307	9,866,884 (0.95%)	34,526	8,474,428 (0.70%)
DNA Transposons	3,697	536,502 (0.05%)	3,561	511,534 (0.04%)
Unclassified Interspersed Repeats	2,561	421,784 (0.04%)	2,482	406,059 (0.03%)
Satellites	2,033	235,306 (0.02%)	1,584	187,049 (0.02%)
Low Complexity & Simple Repeats	142,486	6,275,857 (0.61%)	114,664	5,076,913 (0.42%)
**Totals**	**386,216**	**53,708,206 (5.2%)**	**319,560**	**47,699,295 (4.0%)**

1 Simple, unscaffolded (SMACv1.0) *de novo* assembly (1.035 Gb).

2 Scaffolded (SMACv1.1) *de novo* assembly (1.205 Gb including gaps with N’s).

To further investigate the repetitive content of the scarlet macaw genome, we utilized the program PHOBOS (v3.3.12) [40] to detect and characterize genome-wide tandem repeats (microsatellite loci) within the simple *de novo* assembly (SMACv1.0) for the purpose of identifying loci that could potentially be translated into useful molecular markers for scarlet macaw population studies. Altogether, we identified 2,485,786 tandem repeats consisting of 2 to 10 bp sequence motifs which were repeated at least twice, with the full distribution characterized as follows: 350,157 di-, 468,875 tri-, 461,998 tetra-, 485,422 penta-, 466,685 hexa-, 129,706 hepta-, 87,295 octa-, 23,095 nona-, and 12,553 decanucleotide microsatellites (Table S8). The same analysis conducted on the scaffolded scarlet macaw assembly (SMACv1.1) produced evidence for 2,346,573 tandem repeats as follows: 333,739 di-, 445,677 tri-, 435,955 tetra-, 457,988 penta-, 440,902 hexa-, 122,575 hepta-, 75,793 octa-, 21,830 nona-, and 12,114 decanucleotide microsatellites (Table S9). Similar to the results of our RepeatMasker analyses (Table3), the scaffolding procedure (N’s spanning gaps) results in an underestimate of total genome-wide tandem repeats. Studies are currently underway to evaluate a subset of these markers for applications in molecular ecology and population genetics.

To provide the first description of genome-wide variation for an individual wild-caught scarlet macaw, we investigated the frequency and distribution of putative single nucleotide polymorphisms (SNPs) resulting from biparental inheritance of alternative alleles (heterozygosity) within the repeat-masked simple *de novo* assembly (SMACv1.0; chicken and zebra finch repeat libraries) [1]. Collectively, 951,507 biallelic SNPs (Coverage ≥10X and ≤60X) were predicted, with an estimated average genome-wide density of approximately 1.0 SNP/1.00 Kbp for the autosomes (i.e., Z and W excluded by blastn; Table S10). This estimate is higher than for the domestic turkey [16] and lower than for the zebra finch [17], with our estimate reflecting an overall average genome-wide autosomal distribution [41]–[42] as predicted by blastn. As expected, application of the same SNP detection methods [1] to the repeat-masked scaffolded *de novo* assembly (SMACv1.1, chicken and zebra finch repeat libraries), which contains N’s representing gaps, provided evidence for fewer biallelic SNPs (n = 890,527; Coverage ≥10X and ≤60X; Table S11), and a somewhat lower overall average SNP density. Importantly, similar genome-wide estimates of SNP density have not yet been reported for any other species of Pscittacidae [2] (http://aviangenomes.org/budgerigar-raw-reads/). Likewise, genome-wide SNP variation in flycatchers and chickens has been summarized in terms of pairwise comparisons of genome sequences derived from different individuals, species, or domestic lines [21], [43].

### “In silico” Annotation of the Scarlet Macaw Genome

In the absence of cDNA sequences generated from multiple scarlet macaw tissues, we performed an *in silico* annotation of the scarlet macaw nuclear genome as a first step toward enabling genomics research in this species. Specifically, we used GlimmerHMM [44]–[45] to predict exons within the scarlet macaw scaffolded *de novo* assembly (SMACv1.1), with algorithm training conducted using all annotated chicken genes (*G. gallus* assembly 4.0). The chicken was chosen for training GlimmerHMM because of the superior level of annotation available, as compared with the zebra finch, which is at least partially annotated based on chicken sequences. Thereafter, the resulting exon predictions were filtered using a high-throughput distributed BLAST engine implementing the blastx algorithm [1], [46] in conjunction with a custom database containing all available bird proteins (NCBI non-redundant avian protein sequences), with retention of the E-value top hits to avian proteins. Collectively, this total *in silico* approach produced robust statistical support for 14,405 unique annotation models (see Methods; Table S12). However, the number of unique annotation models that are reported here were based on blastx assignments to unique protein hit definitions (i.e., blastx hits with unique accessions), which is actually a vast underestimate of the total unique models predicted for SMACv1.1 (See Table S12). For example, 2,318 annotation models were predicted for seven specific protein accessions representing non-LTR retrovirus reverse transcriptases and/or reverse transcriptase-like genes (*pol*-like ORFs) that were also predicted in large copy numbers throughout the chicken nuclear genome (Table S12, Genbank Accessions AAA49022.1, AAA49023.1, AAA49024.1, AAA49025.1, AAA49026.1, AAA49027.1 AAA49028.1). Likewise, annotation of multi-copy genes within the established chicken and zebra finch genomes often makes use of naming schemes that include “like” or “similar to” a specific Genbank accession for the purpose of distinguishing one loci or model from another. For our first-generation scarlet macaw *in silico* annotation, the prediction culminates with a blastx hit definition (i.e. Genbank protein accession) representing the highest scoring avian protein curated by NCBI. Therefore, multi-copy loci predicted to encode very similar putative proteins may be assigned to the same specific protein accessions by the blastx procedure. It should also be noted that the absence of genome maps and cDNA sequences to further scaffold and annotate the scarlet macaw genome actually precludes complete *in silico* models for most nuclear genes, especially complex genes encoding many exons that are distributed across large physical distances, and/or genes encoding moderate to large proteins. Nevertheless, our *in silico* approach was successful at identifying scarlet macaw scaffolded contigs that were predicted to possess genes encoding some moderate to large proteins. One such example is the predicted scarlet macaw orthologous sequence for chicken and zebra finch cHz-cadherin (Table S12; Genbank Accessions AAQ82055.1, XP_002196034.1), which was independently predicted in four discrete scaffolded contigs (Table S12) that all comparatively align (blastn) to the expected regions of GGA2 and TGU2 (Table S5).

Comparative investigation of all scarlet macaw contig sequences produced by the *de novo* assembly of multi-platform next generation sequence reads revealed a complete scarlet macaw mitochondrial genome (SMACv1.0 Contig70881 Genbank Accession AMXX00000000; SMACv1.1 Contig20041 Genbank Accession AOUJ00000000). Both assemblies were successful at reconstructing the mitochondrial genome at an average coverage of 113X from >24,000 reads (SMACv1.0 and SMACv1.1). Using the *Aratinga pertinax chrysogenys* mitochondrial genome refseq (GenBank Accession HM640208.1) in conjunction with BLAST (blastn, bl2seq, blastp; http://blast.ncbi.nlm.nih.gov/), we annotated 13 scarlet macaw mitochondrial protein coding genes (*ND1*, *ND2*, *COX1*, *COX2*, *ATP8*, *ATP6*, *COX3*, *ND3*, *ND4L*, *ND4*, *ND5*, *ND6*, *CYTB*) and two ribosomal RNA genes (*12S*, *16S;* SMACv1.1 Contig20041). Analyses using tRNAscan-SE (http://lowelab.ucsc.edu/tRNAscan-SE/) [47] revealed evidence for 20 standard mitochondrial tRNA genes (see Table S12). One tRNA-Phe gene was also manually predicted and annotated. The consensus mitochondrial genome spanned 16,993 contiguous bp and possessed an average GC content of 46.89%.

Despite the apparent utility of our *ab initio* nuclear gene predictions, it should also be noted that our *in silico* approach involving one highly diverged but well-annotated reference species for training GlimmerHMM [44]–[45] was not sufficient to predict either partial or complete gene models for all of the expected genes within the scarlet macaw nuclear genome. For example, previous comparative studies of the avian major histocompatibility complex (MHC) have established expectations for gene content among several bird species [15]–[17], [48]–[51], with our *in silico* approach providing evidence for many (i.e., *CD1*, *TAP2*, *MHC class I*, *MHC class IIA*, *MHC class IIB*, *TRIM27*, *TRIM27.1*, *TRIM7.1*, *TRIM7.2*, *TRIM41*), but not all avian MHC genes previously described and annotated (Table S12). While the limitations of our approach were not surprising, the overall performance was sufficient to facilitate the initiation of a formal genomics research program in the scarlet macaw. Future studies consisting of exhaustive, iterative comparative annotation using the full suite of BLAST tools in conjunction with all available sequence repositories (i.e., Genbank, Ensembl, EMBL, DDBJ, Swiss-Prot) are likely to provide additional comparative evidence for the majority of the expected scarlet macaw gene content, with cDNA sequencing from multiple tissues leading to precise refinement of the *in silico* predictions.

To classify the GlimmerHMM-predicted genomic information content within the scaffolded *de novo* scarlet macaw assembly (SMACv1.1), we performed a functional annotation analysis by mapping the sequences onto relevant classification schemes such as Gene Ontology (GO) terms [52] and Swiss-Prot keywords [53] using the Database for Annotation, Visualization, and Integrated Discovery (DAVID) [54]. Collectively, we identified 194 unique GO terms for biological process, 120 unique terms for molecular function, 75 unique terms for cellular component, and 39 unique Swiss-Prot Protein keywords (Table S13). This analysis provides a detailed ontological classification of the predicted genes and proteins, and may be used for comparative genomic approaches that require identification of scarlet macaw loci and associated pathways that may influence parrot traits of interest. For example, multiple GO terms for biological processes, cellular components, and/or molecular functions include representation from human genes reported to modulate differences in cognitive abilities (i.e., *RORB*, *C1QL3*, *ODZ3, RELN, FMR1, FXR1, NF1,* etc) [55]–[56] as well as longevity (i.e., *POT1*, *AKT1*, *AKT3*, *SIRT1/SIRT2/SIRT6*, etc) [57]–[59] (Table S13). Annotation models for all of these loci except *FXR1*, *AKT1*, *SIRT2* and *SIRT6* were predicted (Table S12), thus providing an opportunity for macaw studies focusing on prioritized candidate genes for longevity and intelligence. Relevant to the observed frequencies of specific GO terms (Table S13), it should be noted that the functional analyses described here are biased towards the annotation of genes that are conserved between the chicken and scarlet macaw, because GlimmerHMM was trained using *G. gallus*, and therefore, caution is necessary when interpreting the frequency distributions of GO Terms and Swiss-Prot Protein Keywords, as well as the fold enrichment scores. Caution regarding this limitation is not specific to SMACv1.1, because in the absence of an exhaustive annotation of all putative genes, some of the frequency and fold change estimates are likely to be wrong. Nevertheless, this limitation does not devalue the ontological classifications, which are useful for enabling comparative genomics.

### Whole-genome Analysis of Divergence

With the advent of high-throughput next generation sequencing technologies and new bioinformatic tools, one of the most intriguing scientific questions to be directed towards the interpretation of new genome sequences is “What makes each species unique?” Several previous avian genome studies have focused on enumerating differences in gene copy number, and the presence or absence of genes or gene families, as prioritized by the underlying biology of the organism under investigation (i.e., no chicken or zebra finch genes encode casein milk proteins, salivary-associated proteins, or tooth enamel proteins as compared to mammals) [15]–[17]. Additionally, these studies have also focused on classical tests of selection, such as *K*_A_/*K*_S_ or *d*_N_/*d*_S_ applied to predicted gene sequences [15]–[17], with one caveat being that not all gene sequences are included in these analyses. While important and informative, these analyses do not jointly consider the noncoding portions of the genome (i.e., proximal gene promoters, noncoding DNA possessing functional regulatory elements including repeats), which have been hypothesized to underlie differences in species-specific genome regulation and traits [60]–[63]. Therefore, we took an alternative approach which utilized all of the produced scarlet macaw contig sequences and the full distribution(s) of blastn data (E-value top hits) generated from the two comparative genome alignments performed (SMACv1.0 versus chicken, *G. gallus* 2.1, with subsequent confirmation using *G. gallus* 3.1; SMACv1.0 versus zebra finch, *T. guttata* 1.1, 3.2.4 assembly). Utilization of the simple *de novo* assembly (SMACv1.0) was desirable because it resulted in a shotgun-like fragmentation of the scarlet macaw genome that is essentially devoid of N’s (i.e. gaps), which in relation to the blastn evalue top hits, provides for fine-scale comparative nucleotide alignments often spanning large portions, the majority, or even the entire length of the contig sequences produced.

**Figure 4:**
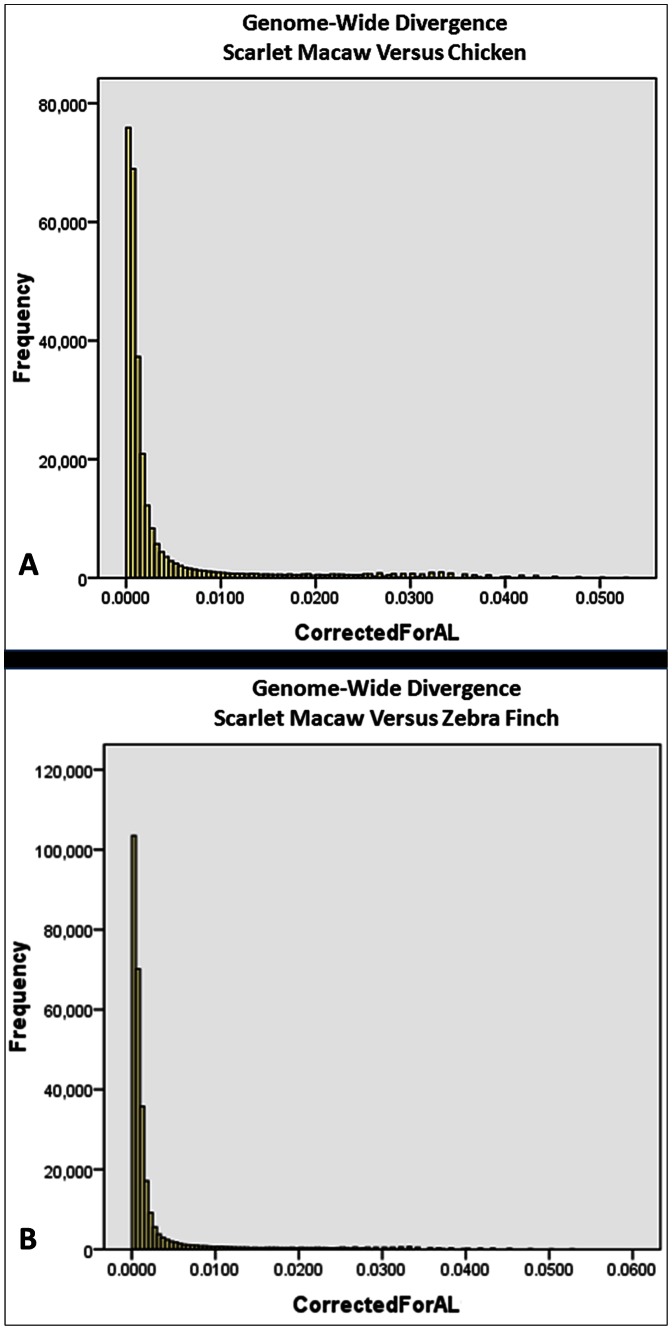
Whole Genome Analysis of Divergence. **(A)** Genome-wide nucleotide-based divergence (CorrectedForAL) between the scarlet macaw (*Ara macao*; simple *de novo* assembly) and chicken genomes (*Gallus gallus* 2.1). **(B)** Genome-wide nucleotide-based divergence (CorrectedForAL) between the scarlet macaw (*Ara macao*; simple *de novo* assembly) and zebra finch genomes (*Taeniopygia guttata* 1.1, 3.2.4). Each histogram represents the full, ordered distribution of the composite variable defined as: 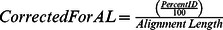. The observed ranges of the composite variable for pane **(A)** and pane **(B)** were 3.89591E-05–0.052631579, and 3.33792E-05–0.052631579, respectively. The left edges of the distributions represent extreme conservation, whereas the right edges indicate extreme putative divergence. Distributional outliers were predicted using a percentile-based approach (99.98^th^ and 0.02^th^) to construct interval bounds capturing >99.9% of the total data points in each ordered distribution.

Briefly, for all scarlet macaw contigs that produced blastn hits to the chicken (n = 282,904) or zebra finch (n = 282,915) genomes (Table S4), we normalized the observed percent identity for differences in alignment length across both comparative genome alignments (see Methods). Evaluation of the corrected percent identity variable (CorrectedForAL; Figure 4) for both genome alignments revealed evidence for non-normal (right skewed) distributions (*P*<0.001 for both alignments, Kolmogorov-Smirnov Test with Lilliefors Correction; Figure 4). Consequently, we took a percentile approach (Percentiles = 99.98% and 0.02%) to establish interval bounds delineating the tails of the ordered distributions, and used this information to identify scarlet macaw contig outliers for extreme nucleotide-based conservation and divergence with respect to the chicken and zebra finch genomes (Figure 4). Validation of macaw contig outlier status was confirmed using the newest chicken genome build (*G. gallus* 3.1) available at the time of analysis. Thereafter, we used the suite of BLAST tools in conjunction with three databases (refseq_genomic; reseq_rna; nr/nt) to characterize the nucleotide sequences of the outlier contigs (see Table S14). Comparison of the scarlet macaw and chicken genomes revealed outlier macaw contigs (Figure 4, Table S14) possessing coding and noncoding loci which were subsequently characterized based on either known function and/or the results of independent human genome wide association studies (GWAS; Table 4) [57], [64]–[110]. Notably, many genic outliers for conservation detected in our analysis (i.e., *TTN*, Mitochondrial genome, *EYA1*) are known to be conserved across a variety of divergent taxa, most likely due to strong purifying selection [111]–[114], with rare deleterious mutations causing disorders, diseases, or molecular dysfunction [109], [113]–[114]. Within the contigs classified as outliers for extreme conservation or divergence, we also observed many noncoding sequences as well (Table S14), as predicted by blastn, thereby supporting the supposition that noncoding genomic regions may underlie differences in species-specific genome regulation and traits [60]–[63]. Interestingly, the sequenced breed of chicken (Red Junglefowl) and the scarlet macaw are very similar in overall body length (excluding tails; similar ‘height’ and head to rump body length) [8], [115]–[116], which is consistent with the observation that macaw contigs containing nucleotide sequences for orthologous human height genes (or their proximal noncoding flanking regions) are among the most highly conserved between the two avian species in our analysis (Table S14). However, even more intriguing was the observation that macaw contigs containing predicted gene sequences (or their proximal noncoding flanking regions) previously associated with human traits such as the acquisition and utilization of speech (*FOXP1*/*FOXP2*-*CNTNAP2*) [74], [80], exceptional longevity (*POT1*) [57], and intelligence (*TRMT1, LHFP*) [84], [87]–[88] were among the most diverged as compared to the chicken genome (Table S14), which is fully concordant with known parrot traits of interest [5]–[7], [18]. We also observed a macaw outlier contig that was predicted to contain a *CDK5RAP2* intron, which is a gene implicated in the developmental manifestation of microcephaly, and in the evolution of brain size among vertebrate species [85]–[86]. This observation is compatible with the fact that macaws possess the largest brain volume (telencephalic) among 154 divergent avian species sampled from 13 families, with estimates of brain volume that are >52% larger than *G. gallus* (Red Junglefowl). Moreover, telencephalic volume in birds has been strongly correlated with measures of social complexity [117].

**Table 4:** SMACv1.0 simple *de novo* outlier contigs from a genome-wide analysis of divergence with the chicken.

Predicted Outlier Contig Genes and Pathways^1^	Known Function or GWAS Trait Classification	References
Mitochondrial Genome	Energy Production	[64]
*SEMA3A*, *RARB*, *PRICKLE1*, *SNW1*	Neuronal Development	[65]–[69]
*SEMA3A, ATXN1, CNTNAP2, INPP5E,*	Neurological Disorders	[70]–[80]
Intergenic *TTLL7* and *LPHN2*, *GPR37*,		
*FOXP1*/*FOXP2*-*CNTNAP2* Pathway		
*ATXN1*	Motor Coordination	[71]
*GPR37*, *ATXN1*, *NRCAM*	Cognition, Learning	[81]–[83]
*ATXN1*, *INPP5E*, *TRMT1*, *ADK*	Intelligence	[72], [77], [84]
*FOXP1*/*FOXP2*-*CNTNAP2* Pathway	Speech	[74], [80]
*CDK5RAP2*	Brain Size	[85]–[86]
*LHFP*	Hippocampal Volume and Intelligence	[87]–[88]
*ADAMTSL3*, *TEAD1*, *EXT1*	Height	[89]–[93]
Intergenic *BTG1*, Intergenic *PRICKLE1*,	Heart Failure	[94]–[95]
*PARVA*		
*VTI1A*, *NFIA*	Heart Ventricular Conduction	[96]
*PTPRG*, *TTN*	Heart Q-wave T-wave Interval Length	[97]
*CAMK4*	Blood Pressure	[98]
Intergenic *WNK1* and *NINJ2*	Stroke	[99]
*CNTNAP2*, *COLEC10*	Bone Mass	[100]–[101]
*UBE2L3*, *THRB*	Blood Traits	[102]
*LIMK2*, *ST6Gal1*	Diabetes	[103]–[104]
*PARD3B*, *CHSY3*	Response or Susceptibility to Viruses	[105]–[106]
*WDR36*, Intergenic *CHD7* and *CLVS1*,	Asthma, Lung Function, Respiratory	[107]–[109]
*TTN*		
*VEGFA*, *COL8A1*	Age-Related Macular Degeneration	[110]
*POT1*	Longevity	[57]

1 For outlier direction, see Table S14.

Application of the same analysis of divergence to a comparison of the scarlet macaw and zebra finch genomes revealed outlier macaw contigs (Figure 4, Table S14) which were predicted to possess coding and noncoding loci that were also characterized according to known function and/or independent human GWAS results (Table 5) [65], [96]–[97], [118]–[159]. Interestingly, some of the same macaw contigs and corresponding gene sequences classified as outliers for conservation in a comparison of macaw to chicken also were detected in the comparison to zebra finch (i.e., *TTN*, *VTI1A*; see Table S14), thereby underscoring the potential biological and developmental importance of these loci across divergent avian lineages. Moreover, both analyses of divergence (Figure 4, Table S14) revealed evidence for extreme nucleotide-based conservation across genes (and/or their proximal intergenic noncoding regions) that have previously been associated with specific developmental processes and trait classes including neuronal development, risk for developing neurological disorders (i.e., autism), cognition, aspects of osteogenesis, brain-specific traits including neuroanatomic features, ocular and cardiovascular traits, and risk factors for diabetes. Perhaps the most interesting result derived from our analysis of divergence between the macaw and the zebra finch genomes was the identification of scarlet macaw outlier contigs (diverged) that aligned to genes, or their proximal noncoding sequences, which have been implicated in oral feeding success in human neonates (*NPY2R*) [155], cerebrovascular developmental disorders such as cavernous malformations (*ZPLD1*) [145], and brain striatal volume (*VPS52*) [137] (Table S14). Importantly, the size of the neostriatum-hyperstriatum ventral complex in birds is known to be a good predictor of feeding innovation, which is considered a measure of cognitive complexity [160]. Additionally, brain striatal volume is predictive of other neuroanatomical measures in mice (i.e., hippocampus) [137] as well as telencephalic brain volume in birds [117]. Therefore, the results of our analyses of divergence (i.e., *VPS52*-divergence; Table S14) are concordant with the observation that macaws possess telencephalic brain volumes that are >21% larger than the songbird *T. guttata* (zebra finch) [117], thereby indicating that *VPS52* should be considered a candidate gene for future studies seeking to elucidate loci involved in the evolution of avian telencephalic brain volume.

**Table 5:** SMACv1.0 simple *de novo* outlier contigs from a genome-wide analysis of divergence with the zebra finch.

Predicted Outlier Contig Genes and Proteins^1^	Known Function or GWAS Trait Classification	References
*EYA2*, *EPHB3*, *BCL6*, *NRP2*, *ALCAM*	Neuronal Development	[118]–[120]
*SATB2*	Neuron Specification	[121]
*ALCAM*, *BCL6*, *NRP2*, *ALDH6A1*, *TP63*, *CUX1*, *ATXN1*,	Neurological Disorders	[120], [122]–[132]
*VIPR2*, *WHSC1*, *BRSK2*, *CLINT1*, *DUSP8*, *ANKFN1*, *PI4KA*		
*ANKFN1*, *LPP*, *BOC*	Human Developmental Anomalies	[133]–[135]
*ALDH6A1*	Hippocampal and Cognitive Aging	[122]
UPF0632 Protein A	White Matter Integrity in Old Age	[136]
*VPS52*	Brian Striatal Volume, Cognition	[136]–[138]
*LDB2*, *DMXL2*	Susceptibility to Coronary Artery Disease	[139]–[140]
*SLC38A1*	Cardiovascular-Left Ventricular Mass	[141]
*ABCB8*, *RBM20*,	Cardiomyopathy	[142]–[143]
*FOXC1*	Embryonic Cardiovascular Development	[144]
*VTI1A*	Heart Ventricular Conduction	[96]
*TTN*	Heart Q-wave T-wave Interval Length	[97]
*ZPLD1*	Cerebrovascular Developmental Disorders	[145]
*FOXF2*	Developmental Disorders of the Genitalia	[146]
*SATB2*	Osteogenic Differentiation, Regeneration	[147]
*MAP3K4*	Myogenesis	[148]
*DCT*	Pigment Biosynthesis	[149]
*VSX2*	Eye Development-Microphthalmia	[150]
*EYA2*	Ocular Neural Pattern Development	[65]
*SCFD2*	Optic Disc Size-Cup Area	[151]
*SDF2L1*	Innate Immunity	[152]
*MKX*	Tendon Differentiation and Development	[153]
*NGEF*	Adiposity	[154]
NPY2R	Feeding Behavior, Oral Feeding Success	[155]
*TCF7L2*, Intergenic *GPATCH2* and *ESRRG*, *FOSL2*, *PPP1CB*	Diabetes	[156]–[159]

1 For outlier direction, see Table S14.

Additional investigations of the concordance between SMACv1.0 and SMACv1.1 revealed several interesting features relative to our whole genome analyses of divergence. Specifically, scarlet macaw simple *de novo* contigs (see Table S14) possessing some of the most interesting coding and noncoding loci previously associated with traits of interest such as intelligence (177250-*TRMT1*; 256909-*LHFP*), the acquisition and utilization of speech (256158-*CNTNAP2*), longevity (42281-*POT1*), height (69396-*EXT1*), hippocampal and cognitive aging (*ALDH6A1*), and telencephalic brain volume (189862-*VPS52*) were all successfully mapped onto the scaffolded assembly, with evidence for very few or zero alignment gaps (Table S3).

In a final analysis of the SMACv1.0 contig sequences which were classified as putative outliers for conservation and divergence (Table S14), we tested for significant differences in the occurrence of putative SNPs (i.e., enrichment) among the two disparate outlier classes (total putative SNPs, total contig bp) for each comparative whole genome analysis of divergence. Only the comparison with the chicken genome produced evidence for a significant difference (conserved vs diverged, *P*<0.000194; chi-square test, 1 df), with the diverged outlier contigs predicted to possess a higher overall density of putative SNP. This result is relatively unsurprising given the larger estimated time since divergence (122–125 MYA; http://www.timetree.org/) [19]–[20] between the scarlet macaw and the chicken, but is also potentially misleading. For example, all putative outlier contigs for divergence produced a 19–22 bp blastn alignment with 100% identity to the chicken or zebra finch genome regardless of contig size, which is actually highly compatible with the supposition that species-specific insertion deletion mutations may be a plausible driving force for achieving outlier status in both analyses (Table S14, Figure 4).

### Quality Control Investigation for Analyses of Divergence

Relevant to our nucleotide-based analyses of divergence (Table S14), it should be noted that all scarlet macaw contigs classified as putative outliers for divergence (Figure 4, right tail) shared one unifying feature: A 19–22 bp alignment with 100% identity to a reference genome (i.e., chicken or zebra finch) regardless of contig size (Range = 208 bp to 1,782 bp; Median = 584 bp; Mean = 651 bp). At least three plausible explanations for this include: 1) The orthologous sequences are simply missing from the chicken and/or zebra finch genome assemblies; 2) The contigs are misassembled; or 3) The contigs represent true outliers for nucleotide-based divergence, including species-specific insertion-deletion mutations. Importantly, we recognize that some sequences are in fact missing from every draft genome assembly. Therefore, we searched three NCBI databases (i.e., refseq_genomic, refseq_rna, nr/nt) for nucleotide alignments that would facilitate contig characterization and/or help refute the diverged outlier status of these contigs, with at least one database that contains unassembled chicken and zebra finch nucleotide sequences, and in all cases found little or no evidence for a better blastn alignment to the chicken or zebra finch genomes. However, some of these contigs actually provide longer, more significant blastn alignments to other vertebrate species, including other avian species, which is not concordant with outlier status (diverged) resulting solely from contig misassembly (See Table S14).

In relation to our nucleotide-based analyses of divergence, we also observed a trend whereby contigs classified as outliers for conservation (Figure 4; extreme left edge) were moderately large in comparison to those classified as outliers for divergence. Therefore, we conducted several quality control analyses that were designed to help determine whether factors other than nucleotide sequence divergence were responsible for our results (Table S14). First, we used summary data from the two comparative genome alignments performed via blastn to estimate pairwise correlations among the following: scarlet macaw contig size (bp), contig percent GC, contig percent identity, and contig alignment length (bp). Moderate correlations between scarlet macaw contig alignment length and contig size were observed with respect to the chicken (r = 0.614, Nonparametric ρ = 0.689) and zebra finch genome alignments (r = 0.680, Nonparametric ρ = 0.740), whereas weak correlations were observed between percent identity and alignment length (r = −0.294, Nonparametric ρ = −0.426; r = −0.159, Nonparametric ρ = −0.234), respectively. All other investigated parameters possessed weaker correlations. This result is important because the two parameters driving our analysis of divergence are the percent identity and alignment length, which together were used to construct a composite variable (CorrectedForAL) that represents percent identity normalized for alignment length across all individual scarlet macaw contigs which produced blastn alignments to the chicken and zebra finch genomes. Next, we applied the same percentile based approach (Percentiles = 99.98% and 0.02%) used in our analyses of nucleotide sequence divergence to examine the full, ordered distribution of scarlet macaw contig sizes, and determined that only 3 contigs (Table S14, zebra finch analysis; contigs 63925, 63319, 55788) were in common with the 162 implicated as outliers for nucleotide-based conservation or divergence (Table S14). This result argues against the proposition of contig size being deterministic for our results (Figure 4, Table S14).

For larger contigs, such as those classified as outliers for conservation, the blastn procedure may produce multiple meaningful alignments, which are appended below the E-value top hit. These appended results include both noncontiguous (i.e., gaps due to insertion-deletion mutations) and less significant comparative alignments. To assess the reliability of utilizing only the top E-value hit as a proxy for larger contigs which may produce multiple, syntenic, noncontiguous hits spanning the overwhelming majority of the contig length (i.e., >85%), we used the additional non-overlapping alignment data (percent identity, alignment length) for these conserved outlier contigs to recalculate our composite variable via summation (Table S15). For all 112 contigs categorized as conserved outliers, the new composite variable only exacerbated the original outlier status (i.e., extreme conservation; see Table S15). Among all scarlet macaw contigs, those classified as outliers for extreme conservation in our comparative analyses actually represent genomic tracks (Table S14) in which extended nucleotide-based conservation persists for the compared species, which cannot occur in the presence of species-specific genomic rearrangements, copy number variants whereby one or more boundaries are traversed, or frequent and complex repetitive elements. Finally, it should also be noted that only contigs which produced blastn results (>99.97%) could be included in our analyses of divergence and quality control investigation, as they provided the data necessary to construct the composite variable. A table of contigs for which no significant alignments were achieved with respect to the chicken or zebra finch genomes is provided in Table S4.

### Conclusions and Future Studies

Similar to the recently published Puerto Rican parrot genome [2], we demonstrate that low-cost (Table 1), high quality (Table 2) draft *de novo* genome assemblies can be generated for avian species that are currently without sophisticated genome maps. Interestingly, our whole-genome analysis of divergence may also be used to comparatively assess genome quality, with the overwhelming majority of the scarlet macaw draft genome assembly exhibiting moderate to high levels of conservation with both the chicken and zebra finch genomes (Table S4, Table S5, Figure 4). Likewise, we also identify regions of the avian genome which are highly conserved across multiple divergent lineages (i.e. predicted genes and noncoding loci), thereby reflecting their likely biological and developmental importance among birds; and simultaneously provide evidence for lineage-specific divergence, with some macaw genes and noncoding regions that coincide with loci implicated by independent human GWAS studies focusing on intelligence and longevity (Table 4, Table 5, Table S14; Figure 4). Therefore, the results of our nucleotide-based analyses of divergence provide prioritized candidate genes and noncoding regions for testing hypotheses related to the evolution of some parrot traits of interest, which are most likely to be modulated by qualitative changes in the products of protein coding genes as well as differences in how avian genomes are regulated within and between lineages.

## Methods

### Source of Scarlet Macaw Genomic DNA

Unlike food animals that are intentionally propagated for slaughter, or model organisms which may be sacrificed within some research settings, the scarlet macaw is a wildlife species for which international conservation and management efforts are currently underway [8]–[14]. Therefore, we utilized whole blood acquired during the routine veterinary medical care provided for a female, wild-caught scarlet macaw (“Neblina”, Blank Park Zoo, Des Moines, IA) to isolate high molecular weight genomic DNA using the UltraClean™ DNA Blood Isolation Kit (MO BIO Laboratories, Inc., Carlsbad, CA). The blood was used with the permission of Blank Park Zoo. The protocol for isolating genomic DNA followed the manufacturer’s recommendations, with the following exception: Because avian red blood cells are nucleated, a total of 25 µl of whole blood was used for each extraction. For each individual extraction we confirmed the presence of high molecular weight genomic DNA by agarose gel electrophoresis, with subsequent quantification of individual isolates performed using a Nano Drop 1000 (Thermo Fisher Scientific, Wilmington, DE).

### Multi-platform Sequencing Strategy

#### Roche 454 titanium sequencing

A whole-genome random shotgun DNA library for sequencing on the Roche 454 GS-FLX instrument was prepared using standard protocols provided by the manufacturer (http://454.com/applications/whole-genome-sequencing). This included nebulization of 5 µg of high molecular weight genomic DNA followed by isolation of fragments between 500–600 bp. This fraction was then subjected to end polishing and repair, random ligation of sequencing adaptors (forward and reverse = Linkers A and B), emulsion PCR, and sequencing on the GS-FLX instrument using titanium reagents as directed by the manufacturer (Roche Applied Science, Indianapolis, IN).

#### Illumina genome analyzer IIx and HiSeq 2000 sequencing

For Illumina mate pair (MP) library preparation, scarlet macaw genomic DNA was first visualized by agarose gel electrophoresis to confirm that high molecular weight fragments were intact and abundant, which is a requirement for optimal MP library construction. Sequencing libraries were created by following the Illumina Mate Pair v2 Library Preparation procedure for 2–5 Kbp fragments (Part #15008135 Rev A; Illumina Inc., San Diego, CA). Briefly, genomic DNA was quantified with the Qubit fluorometer (Life Technologies, Grand Island, NY) and 10 µg was sheared to an average size of either 2.5 Kbp or 5.0 Kbp using the Hydroshear system (Digilab Inc., Holliston, MA). Fragmented DNA was end-repaired using a mix of natural and biotinylated dDNTP’s in the presence of T4 DNA polymerase, T4 polynucleotide kinase, and Klenow DNA polymerase (100 µl volume). The sample was purified using the QIAEX II protocol as described for desalting and concentrating DNA solutions (Qiagen Inc., Valencia, CA), and loaded onto a 0.6% agarose gel (15 hrs at 20 V) for fragment size selection. Gel fragments were excised at average sizes of 2.5 Kbp or 5.0 Kbp and purified using the QIAEX II kit. DNA was quantified using the Qubit fluorometer and a total of 300–600 ng of size-selected DNA was used as input for an intramolecular circularization reaction (30°C, 16 hrs). Circularized DNA was exposed to DNA exonuclease to digest linear fragments, and the enzyme was inactivated prior to sonication (QSonica Inc., Newtown, CT), with an average final size of ≈ 200–500 bp. Following purification using the QIAquick PCR purification kit (Qiagen), the biotinylated DNA was bound to M-280 Streptavidin magnetic beads (Life Technologies) and washed several times. Bead- bound DNA was adapted for Illumina sequencing using standard end repair, A-tailing and ligation steps which join Illumina paired end (PE) oligo adapters to all DNA fragments. Ligated DNA molecules were selectively amplified with 10 cycles of PCR using PE PCR primer 1.0 and 2.0 (Illumina Inc.). The amplified (i.e. no longer bead bound DNA) was size selected on a 2% agarose gel (100 V, 45 min), and final sequencing fragments were excised by selection of 350–650 bp fragments. Following gel purification using the QIAquick gel extraction kit (Qiagen Inc), MP libraries were assessed using the High Sensitivity DNA Bioanalyzer 2100 assay (Agilent technologies), and quantified using the Qubit fluorometer. The final MP libraries were then diluted to 10 nM. Similar procedures were also used for Illumina paired-end (PE) library preparation. Briefly, 5 µg of scarlet macaw genomic DNA was sheared to approximately 300 bp (average size) via sonication, end polished, A-tailed, ligated with standard Illumina PE adapters, size selected to ≈ 350 bp on a 2% agarose gel (120 v 90 min), stained with ethidium bromide for visualization, and PCR amplified for 10 cycles using Illumina PE primers 1.0 and 2.0.

At the onset of this project, the Illumina HiSeq 2000 was not yet available. Therefore, we initially conducted 2×120 small insert PE sequencing on the Illumina GAIIx with standard Illumina Cycle Sequencing kits (v5; Illumina Inc., San Diego, CA) and a final loading concentration of 7.5 pM. Thereafter, we performed 2×100 small insert PE and 2×50 MP sequencing on the Illumina HiSeq 2000 using v2 Illumina Cycle Sequencing kits (Illumina Inc.), and final loading concentrations of 4.1 pM and 6 pM, respectively. All clustering and base-calling (CASAVA-1.7.0; Illumina Inc.) was performed as recommended by the manufacturer.

A summary of trimmed Illumina reads for all libraries is depicted in Table 1.

### Genome Assembly

All sequence reads (Roche 454, Illumina GAIIx, Illumina HiSeq 2000) were first trimmed for quality and adapter sequences within the CLC Genomics Workbench (v4.8). Briefly, the trimming algorithm converts Phred scale quality scores (*Q*) for each base in all reads to an error probability defined as: 
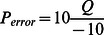
. Therefore, low *P_error_* values indicate high quality bases. Thereafter, for every base a new variable was calculated as: *Limit* - *P_error_*, with the *Limit* value set as 0.05. Notably, this variable will be negative for low quality bases, thereby indicating that the error probability is high. For every base in a given read, and across all imported reads, the Workbench calculates the running sum for this variable. If the sum drops below zero, it is set to zero. The portion of the sequence retained after trimming is the region between the first positive value of the running sum, and the highest value, with everything occurring before and after this region trimmed from every read. A sequence read will be completely discarded if the running sum is never greater than zero. Example calculations using Microsoft Excel are provided in Table S16. At the conclusion of quality trimming, a second algorithm trims ambiguous nucleotides (N) from the ends of every sequence read by referring to a user-specified maximum number of ambiguous nucleotides allowed (n = 3) at each end of the sequence, and subsequently removes all other ambiguous bases. Finally, the Workbench contains an editable library of the most common next-generation sequencing adapters (i.e., Illumina; Roche 454). We used this editable library to identify and select all sequencing adapters that could potentially be present in our multiplatform sequencing reads, and then used the Smith-Waterman alignment algorithm to search every read for user-specified adapters. For every match found, we directed the Workbench to remove the adapter sequence.

For the simple *de novo* assembly, the CLC assembler implements the following general procedures: 1) Creation of a table of “words” observed in the sequence data, with retention and utilization of “word” frequency data; 2) Creation of a de Bruijn graph from the “word” table; 3) Utilization of the sequence reads to resolve paths through bubbles caused by SNPs, read errors, and small repeats; 4) Utilization of paired read information (i.e., paired distances and orientation of reads) to resolve more complex bubbles (i.e., larger repeats and/or structural variation); 5) Output of final *de novo* contigs derived from a preponderance of evidence supporting discrete “word” paths. For the scaffolded *de novo* assembly, the CLC algorithm (v4.9) implemented one additional step in which paired reads spanning two contigs were used to estimate the distance between the contigs, determine their relative orientation, and join them where appropriate using “N’s”; the number of which reflect the estimated intercontig distance. For both assemblies we utilized the same strict, user-specified assembly parameters in conjunction with all trimmed, unmasked sequence reads (Table 1) as follows: add conflict annotations = yes; conflict resolution = vote (A,T,C,G); non-specific matches = ignore; minimum contig length = 500 bp; update contigs based on the mapping back procedure = yes; override specified paired distances = no; mismatch cost = 2; insertion cost = 3; deletion cost = 3; minimum read length fraction = 0.95; minimum fraction of nucleotide identity (similarity) = 0.90. The mapping back process, which also enforces the paired distance and read orientation settings, served two important functions: 1) The potential for fine-scale adjustment of the final (consensus) *de novo* contig sequences; and 2) The removal of contigs and/or structurally suspicious regions that were not well-supported by reference mapping. Relevant to both assemblies, it should be noted that because paired distances within the Workbench are user-specified, incorrect specification of the true paired distances (i.e., too narrow or too wide) negatively impacts *de novo* genome assembly. Therefore, using *a priori* knowledge regarding how each library was constructed and characterized (i.e., agarose gel electrophoresis; Agilent Bioanalyzer) as a guide, we initially assembled the sequence reads multiple times, each with incremental increases in the specified paired distances, until the observed paired distances for each library most closely resembled a bell shaped curve centered about a mean that was compatible with data derived from library construction and assessment. Importantly, all fragment populations used for the construction of sequencing libraries actually represent a range of fragment sizes, and thus paired distances were also expected to vary in this same way. Histograms to assess the observed distribution of paired distances for each library were created and visualized within the CLC Genomics Workbench (v4.8, 4.9). Evidence for user-specified paired distances being too narrow manifested as a partial or severely truncated curve, or a sharp spike without tails within a histogram, whereas those that exhibited extended tails comprised of very low frequency observations were deemed likely to be too wide, thus promoting misassembly. For both scarlet macaw genome assemblies, the user-specified paired distances for all libraries are described in Table 1. Finally, to suppress genome-wide misassembly, the CLC assembler (i.e., simple *de novo*, scaffolded) was instructed to break paired reads exhibiting the wrong distance or orientation(s), and only utilize those reads as single reads within the assembly process. This approach is conservative and favors the creation of more contigs with smaller N50 over the creation of larger and fewer contigs that are likely to contain more assembly errors.

### Estimating Concordance between Assemblies

Treating all scarlet macaw simple *de novo* contig sequences <8,000 bp as individual sequence reads (SMACv1.0), we used the CLC Large Gap Read Mapper algorithm to iteratively search the scaffolded genome assembly (SMACv1.1) for the best matches, with the contig size restriction being a limitation of the mapping algorithm itself (v4.9). Briefly, the mapping workflow for contigs <8,000 bp consisted of a two stage process. In the first iterative mapping analysis, we used the following settings: create report = yes; max hits per segment = 10; minimum read length fraction = 0.50; minimum fraction of identity (similarity) = 0.90; mismatch cost = 2; insertion cost = 3; deletion cost = 3; multi-match mode = ignore; max distance from initial alignment (i.e. seed) = 250 bp. These settings require ≥50% of every simple *de novo* contig <8,000 bp to map with high similarity. Unambiguous mapping data was produced for 207,998 contigs (73.5%). For the second round of mapping, we parsed out the simple *de novo* contig sequences which initially produced no mapping results to create a new input file, reduced the minimum read length fraction to 0.20, but retained all other original settings, and produced unambiguous mapping data for 24,400 contigs (8.6%). For each round of CLC Large Gap Read Mapping, we created a SAM output file, which was used to parse out the coordinates of all mapped simple *de novo* contigs, for the purpose of creating a reference table summarizing the concordance between the two assemblies (Table S3). Thereafter, 31,379 simple *de novo* contigs ≥8,000 bp were successfully mapped onto the scaffolded genome assembly using blastn (version 2.2.24+). Informatic examination of the blastn alignment data provided evidence that the top three hits (E-value) for many contigs were sufficient to cover the majority of the sequence, with some contigs that produced one alignment spanning most of the query sequence. Therefore, we parsed out the top three hits (E-value) for each large contig and joined the results to the reference table (Table S3). All parsing and joining was performed using either Microsoft SQL Server 2008 R2 or custom engineered software.

### Comparative Genome Alignment, Characterization of Repeat Content, and SNP Prediction

The simple *de novo* scarlet macaw genome (SMACv1.0) was aligned to the chicken (*G. gallus* 2.1) and zebra finch (*T. guttata* 1.1, 3.2.4) reference genome assemblies using the blastn algorithm (version 2.2.24+). Scarlet macaw contigs containing interesting features (i.e., outliers from the analysis of divergence) were also assessed using the recently released chicken (*G. gallus* 3.1) and zebra finch genome resources (Updated ChrUn and unplaced), with these updates occurring at the conclusion of this study. To minimize disk space associated with output files while simultaneously enabling continuous data processing beyond blastn searches on a local workstation, we used an E-value step-down procedure as follows: 1) Initial cutoff = 1E-50; 2) Second stage cutoff = 1E-25; 3) Final stage cutoff = None specified, blastn default E-value. After each step, we exported the results and parsed out the top E-value hit for each scarlet macaw contig, and subsequently created a new input file that only contained contigs which produced no blastn results. All parsing, editing, and joining was performed using either Microsoft SQL Server 2008 R2 or custom engineered software. The same workflow was used to align the scaffolded scarlet macaw genome (SMACv1.1) to the chicken (*G. gallus* 4.0) and zebra finch (*T. guttata* 1.1, 3.2.4) reference genomes using the blastn algorithm (version 2.2.26+) with the following exception: 1) Initial cutoff = 1E-20; 2) Second stage cutoff = None specified, blastn default E-value. Blastn results consisting of E-value ties for individual contigs (i.e. identical E-values for the top two or more hits) were almost exclusively limited to E-values of zero, and were broken by bitscore and alignment length.

To estimate the minimum repetitive content within the scarlet macaw genome, all *de novo* contigs (SMACv1.0, SMACv1.1) were processed with RepeatMasker (http://www.repeatmasker.org/; RepBase16.0.1). Briefly, we conducted a two-stage, composite analysis which consisted of masking the scarlet macaw contigs with both the chicken and zebra finch repeat libraries to produce a cumulative estimate of detectable repetitive content. Specifically, we first masked the contigs using the chicken repeat library, and subsequently used the masked contigs as the input file for a second analysis employing the zebra finch repeat library. PHOBOS (v3.3.12) [40] was also used to detect and characterize genome-wide microsatellite loci with the following settings: Extend exact search; Repeat unit size range from 2 to 10; Maximum successive N’s allowed in a repeat = 2; Typical default options for imperfect search; Minimum and maximum percent perfection = 80% and 100%, respectively. Moreover, the average coverage and total number of comparative blastn hits for each *de novo* scarlet macaw contig was also used to provide insight regarding unmasked repeats when cross referenced with the results of RepeatMasker (Tables S4, S5, S6, and S7). However, it should be noted that the overwhelming majority of all scarlet macaw contigs produced ≤5 hits when aligned to the chicken and zebra finch genomes via blastn (Table S4, S5).

Following the two-stage RepeatMasker analysis, the masked (chicken+zebra finch libraries) *de novo* scarlet macaw contigs (SMACv1.0, SMACv1.1) became the reference sequences used for SNP prediction; an approach similar to that used for the rainbow trout [161] and white-tailed deer [1]. After reference mapping all sequence reads to the double-masked assemblies using the same strict assembly parameters described above, a SNP detection analysis employing the Neighborhood Quality Standard algorithm [1], [162]–[163] within the CLC Genomics Workbench (v4.8,4.9) was invoked using the following parameters: annotate consensus = yes, create table = yes, maximum gap and mismatch count = 2, minimum average quality = 20, minimum central quality = 20, minimum coverage = 10X, minimum variant frequency = 35%, SNP analysis window = 11 bp. Custom scripts were developed to parse putative SNP locations from contigs that were aligned to the chicken and zebra finch genomes, and their genomic distribution was subsequently assessed against each avian reference genome.

### Scarlet Macaw Cytogenetics and Zoo-FISH

Detailed protocols for avian metaphase chromosome preparations enabling the reconstruction of karyotypes, including a feather pulp cell culture technique, Giemsa staining, fluorescent *in situ* hybridization (i.e., probes, labeling, hybridization conditions), and microscopy followed those previously described for the California Condor [34]. Moreover, we used the same Zoo-FISH quality control analysis previously reported [34], which involved the application of all chicken chromosome paints to chicken metaphase spreads in order to verify their GGA chromosome of origin (Figure S1). In addition to the sequenced female scarlet macaw, we also acquired metaphase spreads and chromosome counts from feather pulp cell cultures for two additional male scarlet macaws within a local aviary, as previously described [34].

### “In silico” Annotation of the Scarlet Macaw Genome

In the absence of any cDNA sequences for mRNA isolates derived from scarlet macaw tissues, we used GlimmerHMM [16], [44]–[45] to predict putative gene models within the scaffolded *de novo* genome assembly (SMACv1.1; see Dataset S1, Link S1). GlimmerHMM was trained using *G. gallus* 4.0 genes, which is similar to one approach used for annotation of the turkey genome [16]. Thereafter, we used the following approach to predictively define, characterize, and assess support for all annotation models: 1) A custom blast database containing all annotated bird proteins (NCBI refseq; ftp://ftp.ncbi.nlm.nih.gov/refseq/release/vertebrate_other/) was constructed; 2) Using all GlimmerHMM predicted exon sequences in conjunction with a high-throughput distributed blast engine implementing the blastx algorithm [1], [46], we searched the custom protein database for high-quality matches; 3) All top blastx hits with E-values <1E-04 were retained and comprehensively summarized, including the predicted protein sequences, detailed hit definitions, and accession IDs for each hit (Table S12). For the scarlet macaw contig containing the mitochondrion, a blastn search of the NCBI nr/nt database revealed that the *Aratinga pertinax chrysogenys* (Brown-throated Conure) complete mitochondrion had the highest overall identity among all curated sequences. Therefore, using the annotated Conure mitochondrion as a guide (Genbank Accession HM640208), we manually annotated the scarlet macaw mitochondrion using the full suite of available BLAST tools (blastn, bl2seq, blastp; http://blast.ncbi.nlm.nih.gov/), which included 13 protein coding genes (*ND1*, *ND2*, *COX1*, *COX2*, *ATP8*, *ATP6*, *COX3*, *ND3*, *ND4L*, *ND4*, *ND5*, *ND6*, *CYTB*) and two ribosomal RNA genes (*12S*, *16S*). Thereafter, we used tRNAscan-SE (http://lowelab.ucsc.edu/tRNAscan-SE/) to predict tRNA genes. Finally, to classify and visualize the genomic information content derived from the GlimmerHMM-Blastx workflow, we utilized the resulting protein hit definitions and accession numbers (Tables S12) to map the corresponding sequences onto relevant classification schemes such as Gene Ontology (GO) Terms, Swiss-Prot (SP_PIR) keywords, and other available annotation categories using DAVID [52]–[54] (Table S13).

### Whole-genome Analyses of Divergence

For all simple *de novo* scarlet macaw contigs (SMACv1.0) that produced blastn hits to the chicken (n = 282,904) or zebra finch (n = 282,915) genomes, we normalized the observed percent identity for differences in alignment length across both comparative genome alignments using the following formula: 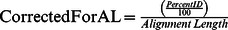. Notably, this method is mathematically related to the p-distance [164], with a previous investigation supporting the use of alignment based sequence comparison and distance estimation for highly conserved genomes [165] (see Tables S4, S15 for evidence of conservation). Thereafter, we visualized the full distribution of this composite variable by producing histograms within the program SPSS Statistics version 17.0 (IBM Corp., Armonk, NY). Likewise, tests of normality (Kolmogorov-Smirnov Test with Lilliefors correction) were also performed in SPSS 17.0, with *P*≤0.01 considered statistically significant. Parametric and nonparametric pairwise correlations for scarlet macaw contig size (bp), contig percent GC, contig percent identity, and contig alignment length (bp) were performed in JMP® Pro version 10.0.0 (Cary, NC). The full distribution for the composite variable “CorrectedForAL” derived from each comparative genome alignment was highly resistant to several standard methods of transformation (i.e., Log; Exponential; etc). Therefore, we used a percentile approach to identify contig outliers based upon establishing interval bounds within the ordered distributions (99.98^th^ and 0.02^th^ percentiles), which captured >99.9% of the total data points in each distribution. Some contigs that displayed evidence of extreme divergence in relation to the chicken and zebra finch genomes possessed identical values for “CorrectedForAL”. The reason for this was that multiple scarlet macaw contigs produced a 19–22 bp alignment with 100% identity to either the chicken or zebra finch reference genome, regardless of contig size. Therefore, the desired percentile cutoff location (i.e., the boundary for extreme divergence) within each distribution fell within a short string of contigs that possessed identical values for “CorrectedForAL” at the edge of the ordered distribution. For this reason, we defined an outlier as any contig which produced a value for “CorrectedForAL” that was either equal to or more extreme than the values corresponding to the 99.98^th^ and 0.02^th^ percentiles. All contigs implicated as outliers were scrutinized by searching three databases curated by NCBI (i.e., refseq_genomic, refseq_rna, nr/nt) for blastn alignments that would further confirm or refute outlier status. For the comparison to chicken, this required searching the newest assembly (*Gallus gallus* 3.1), which was not available at the time of our original analysis; whereas for the comparison with zebra finch, this meant searching the newest releases for ChrUn as well as the unplaced accessions. If a contig originally classified as an outlier for divergence was subsequently observed to produce a longer and more significant alignment, thus firmly placing it within the interval bounds for the specified percentiles, we considered this to be a false positive result and removed it from the list of outliers.

## Supporting Information

Figure S1 **Supplemental microscope images for all chicken-scarlet macaw Zoo-FISH experiments, including the identification of telomeric (TTAGGG) sequences and the 18S, 28S and 5.8S rRNA genes in the scarlet macaw genome, localization of telomeric repeat sequences and inverted DAPI-banding, and lastly, the location of NORs in 3 pairs of microchromosomes (arrows, left) and inverted DAPI-banding (right).**
(PPTX)

Table S1 **Scarlet macaw simple** ***de novo*** **assembly statistics.**
(XLSX)

Table S2 **Scarlet macaw scaffolded** ***de novo*** **assembly statistics.**
(XLSX)

Table S3 **Scarlet macaw simple** ***de novo*** **contig positions in the scaffolded assembly.**
(XLSX)

Table S4 **Summary of the simple** ***de novo*** **scarlet macaw comparative genome alignments using blastn.**
(XLSX)

Table S5 **Summary of the scaffolded** ***de novo*** **scarlet macaw comparative genome alignments using blastn.**
(XLSX)

Table S6 **Summary of repeats predicted in the scarlet macaw simple** ***de novo*** **assembly using RepeatMasker.**
(ZIP)

Table S7 **Summary of repeats predicted in the scarlet macaw scaffolded** ***de novo*** **assembly using RepeatMasker.**
(ZIP)

Table S8 **Summary of scarlet macaw predicted microsatellites within the simple** ***de novo*** **assembly using PHOBOS.** The table was split into two parts (S8a, S8b) to reduce file size.
(ZIP)

Table S9 **Summary of scarlet macaw predicted microsatellites within the scaffolded** ***de novo*** **assembly using PHOBOS.** The table was split into two parts (S9a, S9b) to reduce file size.
(ZIP)

Table S10 **Summary of scarlet macaw putative SNPs predicted within the simple** ***de novo*** **assembly.**
(ZIP)

Table S11 **Summary of scarlet macaw putative SNPs predicted within the scaffolded** ***de novo*** **assembly.**
(ZIP)

Table S12 **Summary of scarlet macaw putative annotation models and corresponding proteins.**
(XLSX)

Table S13 **Summary of scarlet macaw functional annotation analysis using DAVID.**
(XLSX)

Table S14 **Summary of nucleotide-based analyses of divergence**
(DOC)

Table S15 **Summary of conserved outlier quality control analysis.**
(XLSX)

Table S16 **Example of running sum method for quality trimming.**
(XLS)

Link S1 **Scarlet Macaw Genome Project Website includes all supplements plus additional data:** http://vetmed.tamu.edu/schubot/research/scarlet-macaw-genome-project.
(PDF)

Dataset S1 **GlimmerHMM predictions (Zipped File Size 287 MB) are available on the genome project website **(http://vetmed.tamu.edu/schubot/research/scarlet-macaw-genome-project).
(PDF)
