# Supplementary material for: A Multi-Platform Draft de novo Genome Assembly and Comparative Analysis for the Scarlet Macaw (Ara macao)
Source: PLoS One. 2013 May 8;8(5):e62415. doi: 10.1371/journal.pone.0062415 (PMC3648530; doi:10.1371/journal.pone.0062415)
Supplement: Table S14 — Summary of nucleotide-based analyses of divergence (DOC) [file pone.0062415.s015.doc]

**Table S14-A.** SMACv1.0 *de novo* outlier contigs from a genome-wide analysis of divergence with chicken

| **Macaw**  **Contig^1^** | **Outlier Direction^2^** | **Predicted Content^3^** | **Predicted Description^4^** |
| --- | --- | --- | --- |
| 62050 | Conserved | *TTN* | (I,E) Titin (Cardiomyopathy, Dilated 1G) |
| 70881 | Conserved | Mitochondria | Complete Annotated Genome (13 Protein Coding Genes, 21 tRNA Genes, 2 rRNA Genes) |
| 64025 | Conserved | *PTPRG* | (I) Protein Tyrosine Phosphatase Receptor |
| 72877 | Conserved | *LOC100190426* | (I,E) Ribosomal Protein L23 (*RPL23*) |
| 66891 | Conserved | *ERGIC1* | (I,E) ER-Golgi Intermediate Compartment Protein |
| 69396 | Conserved | *EXT1* | (I,E) Exostosin 1 (*LOC100858205*; NW_003763691.1) |
| 104542 | Conserved | Noncoding | Between Thyroid Hormone Receptor Beta (*THRB*) and Retinoic Acid Receptor Beta (*RARB*) **^5^** |
| 64793 | Conserved | *ADORA2A* | (I,E) Adenosine A2A receptor |
| 69895 | Conserved | Noncoding | Between Chromodomain-helicase-DNA-binding Protein 7 (*CHD7*) and Clavesin 1 (*CLVS1*) **^5^** |
| 57264 | Conserved | Noncoding | Between Ephrin Type-A Receptor 7 (*EPHA7*) and Mitogen-activated Protein Kinase 7 (*MAP3K7*) **^5^** |
| 82575 | Conserved | Noncoding | Between Dual Specificity Protein Phosphatase 6 (*DUSP6*) and WD-repeat-protein 51B (*POC1B*) **^6^** |
| 60853 | Conserved | *CLASP1* | (I,E) Cytoplasmic linker associated protein 1 |
| 66256 | Conserved | *VEGFA* | (I,E) Vascular Endothelial Growth Factor A |
| 87385 | Conserved | *BMPR2* | (I,E) Bone Morphogenetic Protein Receptor, Type 2 |
| 107143 | Conserved | *EYA1* | (I,E) Eyes Absent Homolog 1 (*Drosophila* Homolog) |
| 75998 | Conserved | *LOC100226084* | (I,E) similar to family with sequence similarity 51, member A1 (*GEMIN8*) |
| 62662 | Conserved | *HLF* | (I) Hepative Leukemia Factor-like |
| 69742 | Conserved | *ZNF318* | (I,E) Zinc Finger Protein 318 |
| 69271 | Conserved | *LOC100231815* | (I,E) Similar to Riken cDNA |
| 97440 | Conserved | *PARD3B* | (I) Partitioning Defective 3 Homolog B |
| 112754 | Conserved | Noncoding | Between Myosin Regulatory Light-Chain 2B-cardiac (*MYL10*) and *LOC417507* (uncharacterized) **^5^** |
| 100633 | Conserved | *SACS* | (I,E) Spastic Ataxia of Charlevoix-Saquenay (sacsin) |
| 71766 | Conserved | Noncoding | Between TBC1 Domain Family Member 5 isoform-2 (*TBC1D5*) and DNA-binding homeobox (*SATB1*) **^5^** |
| 63201 | Conserved | *LRCH3* | (I,E) Leucine-Rich Repeats and Calponin Homology Domain 3 |
| 85582 | Conserved | Noncoding | Between Uncharacterized *LOC771030* and Ras-GEF Domain-containing member-1c (*RASGEF1C*) **^5^** |
| 50089 | Conserved | *NRCAM* | (I,E) Neuronal Cell Adhesion Molecule |
| 64685 | Conserved | *MFAP3* | (I,E) Microfibril-associated Glycoprotein 3 |
| 65079 | Conserved | *AMOT* | (I,E) Angiomotin P130 |
| 75308 | Conserved | Noncoding | Between *ATXN1* and *LOC770160* (Uncharacterized), Same General Local as contig 104302**^5^** |
| 63731 | Conserved | *ADAMTSL3* | (I,E) ADAMTS-like 3 (a disintegrin-like, metalloprotease domain with thrombospondin motifs-like 3) |
| 69843 | Conserved | *ADK* | (I,E) Adenosine Kinase (Adenosine 5'-phosphotransferase) |
| 71343 | Conserved | *C7orf10* | (I) caiB/baiF CoA-transferase Family Protein C7orf10 |
| 94654 | Conserved | *SUMO1* | (I,E) Small Ubiquitin-related Modifier 1 Precursor |
| 56943 | Conserved | *BTG1* | (I,E) B-cell Translocation Gene 1, Anti-proliferative |
| 80389 | Conserved | Noncoding | Between Transmembrane protein 182 (*TMEM182*) and Uncharacterized Gene (*LOC100857256*)**^5^** |
| 73283 | Conserved | *WNK1,NINJ2* | (I,E) Low Quality Protein Serine/Threonine-Protein Kinase, And Ninjurin 2**^5^** |
| 63362 | Conserved | Noncoding | Between Tubulin Polyglutamylase TTLL7 (*TTLL7*) and Latrophilin 2 (*LPHN2*)**^5^** |
| 64110 | Conserved | *ZNF521* | (I) Zinc Finger Protein 521 |
| 56736 | Conserved | Noncoding | Primarily Between Transcriptional Enhancer Factor TEF-1 (*TEAD1*) and Alpha-Parvin (*PARVA*)**^5^** |
| 59884 | Conserved | *SPECC1L* | (I,E) Cytospin-A, Sperm Antigen With Calponin Homology and Coiled-Coil Domains 1-like |
| 71653 | Conserved | *VTI1A* | (I) Vesicle Transport Through Interaction with t-SNAREs Homolog 1A, Ventricular Conduction |
| 51013 | Conserved | *AHRR* | (I) Aryl Hydrocarbon Receptor Repressor |
| 61807 | Conserved | Noncoding | Between Dual Specificity Phosphatase 1 (*DUSP1*) and ER-Golgi Intermediate Protein (*ERGIC1*)**^5^** |
| 90651 | Conserved | *SFPQ* | (I,E) Splicing Factor Proline / Glutamine Rich |
| 70240 | Conserved | *SEMA3A* | (I,E) Sema Domain, Immunoglobulin Domain, Short Basic Domain, Secreted, (semaphorin) 3A |
| 88512 | Conserved | Noncoding | Between Zinc Finger and BTB Domain 20 (*ZBTB20*) and Dopamine Receptor D3 (*DRD3*)**^5^** |
| 65283 | Conserved | Noncoding | Between Hypermethylated in Cancer 2 (*HIC2*) and Ubiquitin-conjugating Enzyme E2 L3 (*UBE2L3*)**^5^** |
| 88233 | Conserved | *LOC100506330* | (I,E) Outcome Predictor in Acute Leukemia 1 Isoform 3 (No HUGO ID) |
| 52436 | Conserved | Noncoding | Between Uncharacterized Genes (LOC768429) and (LOC768700)**^5^** |
| 78675 | Conserved | *DET1* | (I,E) De-etiolated Homolog 1 (*Arabidopsis*) |
| 91686 | Conserved | *CERS3* | (I,E) Ceramide synthase 3, Previously Known as LAG1 Longevity Assurance Homolog 3 |
| 55306 | Conserved | Noncoding | Between Signal Peptide, CUB Domain, EGF-like 1(*SCUBE1*) and Metallophosphoesterase 1 (*MPPED1*)**^5^** |
| 74301 | Conserved | *COLEC10* | (I,E) Collectin Sub-family Member 10 (C-type lectin) |
| 63562 | Conserved | Two Genes | Phosphoinositide-3-kinase Interacting Protein 1 (*PIK3IP1*) and LIM Domain Kinase 2 (*LIMK2*)**^5^** |
| 101667 | Conserved | *MAPKAPK3* | (I,E) Mitogen-activated Protein Kinase-activated Protein Kinase 3 |
| 52927 | Conserved | *NFIA* | (I,E) Nuclear Factor 1 A-Type, Nuclear Factor I /A |
| 2158 | Diverged | Noncoding | No Repeats, ZF ChrUn (NW_002214813.1) Provides Best Evidence of Putative Orthology |
| 41753 | Diverged | Noncoding | No Repeats, Unknown Orthology |
| 254145 | Diverged | Unknown | High Repeats (ERV), Best Hit 67% ID to Murine Leukemia Virus (AY818896.1), Unknown Orthology |
| 253441 | Diverged | Noncoding | No Repeats, Unknown Orthology |
| 252871 | Diverged | Noncoding | No Repeats, Unknown Orthology |
| 256909 | Diverged | Noncoding | Moderate Repeats (ERV), Between ZF KIAA2032 (*PROSER1*) and *LHFP* (in NW_002197691.1) **^5^** |
| 255850 | Diverged | Unknown | No Repeats, Unknown Orthology, Short (Best) Hit to *ITPR3* (NW_003573525.1) in Elephant |
| 212165 | Diverged | Noncoding | No Repeats, *Ara ararauna* Tandem Repeat P1 Monomer (S48542.1), Unrecognized by RepeatMasker |
| 40975 | Diverged | Noncoding | No Repeats, Unknown Orthology, Several Very Short Hits to Pig, Opossum, and Cow Genomic Scaffolds |
| 257376 | Diverged | Noncoding | No Repeats, ZF ChrUn (NW_002211919.1) and Clawed Frog Unplaced Scaffold (NW_003163790) |
| 258246 | Diverged | Noncoding | No Repeats, Unknown Orthology |
| 256158 | Diverged | *CNTNAP2* | (I) No Repeats, Best Hit to ZF KIAA0868 Intron (NW_002198269.1), 72% ID Across 317 bp |
| 256437 | Diverged | Noncoding | No Repeats, Unknown Orthology, Short Hit to ZF ChrUn (NW_002221201.1) |
| 253773 | Diverged | Noncoding | Low Repeats (ERV), Swan Goose Microsatellite (GU130948.1) |
| 21579 | Diverged | Noncoding | No Repeats, Unknown Orthology |
| 39464 | Diverged | Noncoding | No Repeats, Between *MRPS2* and *INPP5E* (pharbin) in ZF (NW_002197454.1) **^5^** |
| 282453 | Diverged | Noncoding | No Repeats, Unknown Orthology |
| 37475 | Diverged | *CDK5RAP2* | (I) No Repeats, Best Hit to Intron of *CDK5RAP2* in ZF (NW_002197453) 73% ID Across 404 bp |
| 42007 | Diverged | Noncoding | No Repeats, Between *PRICKLE1* and *ADAMTS20* in ZF (NW_002197556.1) 69% ID Across 530 bp**^5^** |
| 177250 | Diverged | *TRMT1* | (I,E) No Repeats, Hits > 8 Species’ mRNAs (Exon) with Putative Intron Also (Top Hit, XM_002724176.1) |
| 281345 | Diverged | Noncoding | No Repeats, Between *GPR35* and *ST6GAL1* in ZF (NW_002199035.1) 72% ID Across 513 bp**^5^** |
| 244522 | Diverged | *SRRT* | (E) No Repeats, Exon Hit in Carolina anole and Others (Top Hit XM_003229455.1) 80% ID Across 184 bp |
| 21731 | Diverged | Noncoding | No Repeats, Unknown Orthology |
| 255840 | Diverged | Noncoding | No Repeats, Unknown Orthology |
| 30816 | Diverged | Noncoding | No Repeats, Between *MOCS2* and *FST* in ZF (NW_002234465.1) 67%-75% ID Across 548 bp**^5^** |
| 213886 | Diverged | Noncoding | High Repeats (ERV), Between *COL8A1* and *URB1* in ZF (NW_002197713.1); Chicken Genes Syntenic |
| 257613 | Diverged | Unknown | High Repeats (ERV), ZF *SLC5A12* mRNA (77% ID, 95 bp; XM_002190396.1), TGU7 Noncoding Also |
| 48748 | Diverged | Unknown | No Repeats, Unknown Orthology |
| 247873 | Diverged | Noncoding | No Repeats, *Ara ararauna* Tandem Repeat P1 Monomer (S48542.1), Unrecognized by RepeatMasker |
| 197926 | Diverged | *LOC100219532* | (E) No Repeats, ZF Exon of Hypothetical Protein (NW_002199036) 73% ID across 120 bp |
| 250743 | Diverged | Noncoding | No Repeats, Unknown Orthology |
| 42281 | Diverged | Noncoding | No Repeats, Between *POT1* and *GPR37* in ZF (NW_002197556.1) 79% ID Across 509 bp**^5^** |
| 146756 | Diverged | Unknown | No Repeats, Between *ANGPTL2* and *FBXW2* in Turkey (NC_015029.1) LOC100228858 in ZF**^5^** |
| 212139 | Diverged | Noncoding | No Repeats, Unknown Orthology |
| 211983 | Diverged | Unknown | No Repeats, Unknown Orthology, (100% Coverage, 77% ID to the filarial nematode *Loa loa*). |
| 214485 | Diverged | Noncoding | No Repeats, Unknown Orthology |
| 253245 | Diverged | Noncoding | No Repeats, Between Hypothetical *LOC100219170* and *LOC100228830* in ZF (NW_002197250)**^5^** |
| 2875 | Diverged | Noncoding | No Repeats, Unknown Orthology |
| 1016 | Diverged | Noncoding | No Repeats, Between *WDR36* and *CAMK4* in ZF (TGUZ; NW_002234456.1)**^5^** |
| 282049 | Diverged | Noncoding | No Repeats, Unknown Orthology |
| 249465 | Diverged | Noncoding | No Repeats, Unknown Orthology, (100% Coverage, 75% ID to the filarial nematode *Loa loa*). |
| 2160 | Diverged | Noncoding | No Repeats, Unknown Orthology |
| 255648 | Diverged | Noncoding | No Repeats, Unknown Orthology |
| 273654 | Diverged | Noncoding | No Repeats, Flanking *CHSY3* in ZF (TGUZ; NW_002234450) |
| 15242 | Diverged | Noncoding | No Repeats,, Between *SNW1* and *LOC100229886* in ZF (NW_002197199.1), Putative Breakpoint |
| 211716 | Diverged | Noncoding | No Repeats, Unknown Orthology |
| 211113 | Diverged | Noncoding | No Repeats, Unknown Orthology |
| 257923 | Diverged | Noncoding | No Repeats, Unknown Orthology |
| 257637 | Diverged | Unknown | No Repeats, Potential provirus ancestral Pol protein (XM_002189835.1) or Noncoding (NW_002212531) |
| 250090 | Diverged | Unknown | No Repeats, ≥10% Coverage *FSTL4* (NT_176338.1) and *CEL* (XM_002924371) |
| 214797 | Diverged | Noncoding | No Repeats, Unknown Orthology |
| 212474 | Diverged | Noncoding | No Repeats, Unknown Orthology |

**^1^** Scarlet macaw contig ID from the simple *de novo* assembly (SMACv1.0).

**^2^** The direction of the outlier in the full blastn distribution for the comparative genome alignment with chicken.

**^3^** Concise prediction (top blastn hit) of the genomic information content for each contig (gene symbol, noncoding, or unknown).

**^4^** Detailed description of the genomic information content for each contig, as evidenced by blastn searches of refseq_genomic, reseq_rna, and nr/nt, with repeat content predicted by RepeatMasker. Outliers for conservation were annotated based on the Chicken Genome. (I) indicates intron(s), (E) indicates exon(s), and (I, E) indicates both. Note, the blast databases are dynamic, and therefore, descriptions correspond to results achieved at the time of analysis (Chicken 3.1 and Zebra Finch Build 1). ZF denotes Zebra Finch.

**^5^** Genes are predicted to be syntenic and proximal in both the chicken and zebra finch genomes via blastn and/or NCBI Map Viewer.

**^6^** Synteny and proximity of genes could not be conclusively determined using the chicken and zebra finch genome resources.

**Table S14-B.** SMACv1.0 *de novo* outlier contigs from a genome-wide analysis of divergence with zebra finch

| **Macaw**  **Contig^1^** | **Outlier Direction^2^** | **Predicted Content^3^** | **Predicted Description^4^** |
| --- | --- | --- | --- |
| 64190 | Conserved | *LOC100227279* | (I/E) Similar to Homeodomain Interacting Protein Kinase 2 (*HIPK2*) |
| 64241 | Conserved | Noncoding | Between Forkhead Box P1 (*FOXP1*) and LOC100231312 (Similar to *GPR27*)**^5^** |
| 89590 | Conserved | *MAD4 (MXD4)* | (I/E) Max Dimerization Protein 4 |
| 78490 | Conserved | *MKX* | (I/E) Similar to Mohawk Homeobox |
| 86961 | Conserved | Noncoding | Between LOC100227593 (Vesicle Transport-tSNAREs 1A, *VTI1A*) and LOC100229459 (*TCF7L2*, T-cell)**^5^** |
| 62050 | Conserved | *TTN* | (I/E) Titin (Cardiomyopathy, Dilated 1G) |
| 70187 | Conserved | Noncoding | Between *LOC100221365* (*MYL10*) and *LOC100219491* (*CUX1*)**^5^** |
| 104302 | Conserved | Noncoding | Between RNA Binding Motif protein 24 (*RBM24*) and Ataxin 1 (*ATXN1*)**^5^** |
| 63925 | Conserved | *LOC100221779* | (I) Uncharacterized Protein Coding Gene Supported by one mRNA |
| 50985 | Conserved | *LOC100232820* | (I) Similar to Sec1 Family Domain Containing 2 (*SCFD2*) |
| 70407 | Conserved | Noncoding | Between *LOC100227330* (*NGEF*) and *LOC100221507* (Similar to *APG16*; HUGO, *ATG16L1*)**^5^** |
| 69572 | Conserved | *LOC100225506* | (I/E) Family with sequence similarity 13 (*FAM13A1)*, member A1(HUGO, *FAM13A*) |
| 68010 | Conserved | *LOC100228483* | (I/E) Uncharacterized Protein Coding Gene Supported by one mRNA |
| 68645 | Conserved | *LOC100226042* | (I/E) Prion Protein Interacting Protein (*PRNPIP*) Gene (HUGO, *ERI3*) |
| 69335 | Conserved | Noncoding | Between Ephrin type-B Receptor 3 (*EPHB3*) and SUMO1/Sentrin Specific Peptidase 5 (*SENP5*)**^5^** |
| 87001 | Conserved | Noncoding | Between *ZFAND6* and *BCL2A1***^5^** |
| 98652 | Conserved | *LOC100227775* | (I/E) Nuclear Receptor Subfamily 5, Group A, Member 2 (*NR5A2*) |
| 68856 | Conserved | *LOC100219562* | (I/E) Similar to PR Domain Containing 1, with ZNF Domain (*PRDM1*) |
| 65216 | Conserved | Noncoding | Between *LOC100225046* (*LPP*) and *LOC100222134* (*BCL6*)**^5^** |
| 73377 | Conserved | *LOC100226701* | (I/E) Mitogen-Activated Protein Kinase 4 (*MAP3K4* ) |
| 61698 | Conserved | Noncoding | Between *LOC100229999* (*VIPR2*) and zinc finger *LOC100232017* (*ZMYND11*)**^5^** |
| 59560 | Conserved | *LOC100222807* | (I/E) Wolf-Hirschhorn Syndrome Candidate 1 (*WHSC1*) |
| 71395 | Conserved | *LOC10023049*8 | (I) Dopachrome Tautomerase (Dopachrome delta-isomerase, Tyrosine-related Protein 2), (*DCT*) |
| 56506 | Conserved | Noncoding | Between *LOC100228731* (*GPATCH2*) and *LOC100219115* (*ESRRG*)**^5^** |
| 72185 | Conserved | Noncoding | Between *LOC100229554* (Hypothetical Protein, mRNA) and LOC100222945 (Pol-like protein ENS-3)**^6^** |
| 80338 | Conserved | *LOC100223978* | (I/E) Similar to Brain-selective kinase 2 (*BRSK2*) |
| 55788 | Conserved | Noncoding | Between *LOC100220955* (*STX16*) and *LOC100227714* (*APCDD1L*)**^5^** |
| 64161 | Conserved | Noncoding | Between *LOC100232015* (*RNF145*) and *LOC100226122* (Epsin 4, HUGO *CLINT1*)**^5^** |
| 69022 | Conserved | Noncoding | Between Zinc Finger Protein 516 (*ZNF516*) and Teashirt Zinc Finger Homeobox 1 (*TSHZ1*)**^5^** |
| 108417 | Conserved | Noncoding | Between Dual Specificity Phosphatase 8 (*DUSP8*) and Riken cDNA 2700078K21 (*MOB2*)**^5^** |
| 95108 | Conserved | Noncoding | Between Neuropeptide VF (*NPVF*) and Nuclear Factor Erythroid-derived 2-like 3 (*NFE2L3*)**^6^** |
| 66135 | Conserved | Noncoding | Between UPF0632 Protein A (*LOC388630*; No HUGO) and Spermatogenesis Associated 6 (*SPATA6*)**^5^** |
| 73796 | Conserved | Noncoding | Between SEC61 gamma subunit (*SEC61G*) and Epidermal Growth Factor Receptor (*EGFR*)**^5^** |
| 63042 | Conserved | Noncoding | Between DMX-Like 2 (*DMXL2*) and Hypothetical *LOC100231185***^5^** |
| 63562 | Conserved | Two Genes | (I/E) LIM Domain Kinase 2 (*LIMK2*) and Phosphoinositide-3-kinase Interacting Protein 1 (*PIK3IP1*)**^5^** |
| 63319 | Conserved | Noncoding | Between INO80 Complex Subunit D (*INO80D*) and Neuropilin 2 (LOC100231962; HUGO *NRP2*)**^5^** |
| 75634 | Conserved | *SATB2* | (I/E) Multiple Introns and Exons of SATB Homeobox 2 |
| 84513 | Conserved | *BOC* | (I) Intron of *BOC* Homolog in Mouse |
| 56770 | Conserved | *ANKFN1* | (I/E) Ankyrin-repeat and Fibronectin Type III Domain Containing 1 (*ANKFN1*) |
| 78419 | Conserved | Noncoding | Between Forkhead Box C1 (*FOXC1*) and Forkhead Box F2 (*FOXF2*)**^6^** |
| 51649 | Conserved | Noncoding | Between SATB Homeobox 2 (*SATB2*) and Phospholipase C-like 1 (*PLCL1*)**^5^** |
| 67753 | Conserved | *PAX2* | (I/E) Paired Box Protein 2, isoforms 1 and 2 |
| 64021 | Conserved | Noncoding | Between Hypermethylated in Cancer 2 (*HIC2*) and PIK4CA Variant Protein (*PI4KA*)**^5^** |
| 75156 | Conserved | Noncoding | Between Topoisomerase (DNA) III beta (*TOP3B*) and Stromal Factor 2-like 1 (*SDF2L1*)**^5^** |
| 64218 | Conserved | *RBM20* | (I/E) RNA Binding Motif Protein 20 |
| 82707 | Conserved | Noncoding | Between Tumor Protein p63 (*TP63*) and LOC100225046 (*LPP*)**^5^** |
| 68180 | Conserved | Noncoding | Between Family With Sequence Similarity 84, B (*FAM84B*) and RIKEN cDNA 0910001A06 (*FAM49B*)**^5^** |
| 72837 | Conserved | *INTS10* | (I/E) Integrator Complex Subunit 10 |
| 60853 | Conserved | *CLASP1* | (I/E) Cytoplasmic linker associated protein 1 |
| 80389 | Conserved | Noncoding | Between Mitochondrial Ribosomal Protein 9 (*MRSP9*) and *LOC100227672*, Flanking *TMEM182***^5^** |
| 70772 | Conserved | Noncoding | Mostly Between Aldehyde Dehydrogenase Family 6-A1 (*ALDH6A1*) and Visual System Homeobox 2 (*VSX2*)**^5^** |
| 81772 | Conserved | *LOC100230191* | (I/E) Hypothetical Protein in ZF (*LOC100230191*), Transmembrane Protein 108 in Chicken (*TMEM108*) |
| 92325 | Conserved | *EYA2* | (I) Eyes Absent Homolog 2 |
| 82706 | Conserved | *SLC38A1* | (I/E) Solute Carrier Family 38, Member 1 |
| 68290 | Conserved | Noncoding | Between FOS-like Antigen 2 (*FOSL2*) and Protein Phosphatase 1, Catalytic Subunit, Beta (*PPP1CB*) |
| 93935 | Conserved | *LDB2* | (I/E) LIM Domain Binding 2 |
| 42698 | Diverged | Unknown | (I/E) No Repeats, Pig *SLC23A3* mRNA, Intron (XM_001925526.2; NC_010457.4), Unknown Orthology |
| 247550 | Diverged | Noncoding | No Repeats, Between *RBM46* and *NPY2R* in Turkey (NC_015014.1) and Chicken (NC_006091.3)**^5^** |
| 210971 | Diverged | Noncoding | No Repeats , Unknown Orthology |
| 256067 | Diverged | Noncoding | No Repeats , Unknown Orthology |
| 48145 | Diverged | Noncoding | No Repeats , Unknown Orthology |
| 270288 | Diverged | Unknown | No Repeats , Unknown Orthology, ≥10% coverage to Cow and Elephant *ABCB8* including mRNAs**^7^** |
| 254146 | Diverged | Noncoding | Low Repeats (Low Complexity), Unknown Orthology |
| 255478 | Diverged | Noncoding | No Repeats , Between *ZPLD1* and *LOC769645*/CD166 antigen-like (*ALCAM*) in Chicken and Turkey**^5^** |
| 42203 | Diverged | Unknown | No Repeats , Unknown Orthology |
| 241876 | Diverged | Unknown | No Repeats , Unknown Orthology |
| 253342 | Diverged | Noncoding | No Repeats , Unknown Orthology |
| 189862 | Diverged | *VPS52* | (I/E) No Repeats , *VPS52* mRNA alignment to Guinea Pig, Tilapia, Horse, and Macaque |
| 256763 | Diverged | Noncoding | No Repeats , Unknown Orthology |
| 38658 | Diverged | Noncoding | No Repeats , Unknown Orthology |

**^1^** Scarlet macaw contig ID from the simple *de novo* assembly (SMACv1.0).

**^2^** The direction of the outlier in the full blastn distribution for the comparative genome alignment with zebra finch.

**^3^** Concise prediction (top blastn hit) of the genomic information content for each contig (gene symbol, noncoding, or unknown).

**^4^** Detailed description of the genomic information content for each contig, as evidenced by blastn searches of refseq_genomic, reseq_rna, and nr/nt, with repeat content predicted by RepeatMasker. Outliers for conservation were annotated based on the Zebra Finch (ZF) Genome. (I) indicates intron(s), (E) indicates exon(s), and (I, E) indicates both. Note, the blast databases are dynamic, and therefore, descriptions correspond to results achieved at the time of analysis (Chicken 3.1 and Zebra Finch Build 1).

**^5^** Genes are predicted to be syntenic and proximal in both the chicken and zebra finch genomes via blastn and/or NCBI Map Viewer.

**^6^** Synteny and proximity of genes could not be conclusively determined using the chicken and zebra finch genome resources.

**^7^** Short alignment to *ABCB8* mRNA with extension into an adjacent intron for Cow (NC_007302.5) and Elephant (NW_003573432).
