# Supplementary material for: A Multi-Platform Draft de novo Genome Assembly and Comparative Analysis for the Scarlet Macaw (Ara macao)
Source: PLoS One. 2013 May 8;8(5):e62415. doi: 10.1371/journal.pone.0062415 (PMC3648530; doi:10.1371/journal.pone.0062415)
Supplement: Link S1 — Scarlet Macaw Genome Project Website includes all supplements plus additional data: http://vetmed.tamu.edu/schubot/research/scarlet-macaw-genome-project. (PDF) [file pone.0062415.s018.pdf]

Please go to the following site for supplementary information:

http://vetmed.tamu.edu/schubot/research/scarlet-macaw-genome-project
